# Exploring the dynamics of flow attenuation at a beaver dam sequence

**DOI:** 10.1002/hyp.14735

**Published:** 2022-11-01

**Authors:** Hugh A. Graham, Alan K. Puttock, Mark Elliott, Karen Anderson, Richard E. Brazier

**Affiliations:** ^1^ Centre for Resilience in Environment, Water and Waste (CREWW), Geography College of Life and Environmental Sciences, University of Exeter Exeter UK; ^2^ Devon Wildlife Trust Exeter Devon UK; ^3^ Environment and Sustainability Institute University of Exeter, Penryn Campus Penryn Cornwall UK

**Keywords:** beaver, beaver dams, *Castor fiber*, ecosystem engineers, floodplain storage, flow attenuation, hydrology, natural flood management

## Abstract

Beavers influence hydrology by constructing woody dams. Using a Before After Control Impact experimental design, we quantified the effects of a beaver dam sequence on the flow regime of a stream in SW England and consider the mechanisms that underpin flow attenuation in beaver wetlands. Rainfall‐driven hydrological events were extracted between 2009 and 2020, for the impacted (*n* = 612) and control (*n* = 634) catchments, capturing events 7 years before and 3 years after beaver occupancy, at the impacted site. General additive models were used to describe average hydrograph geometry across all events. After beaver occupancy, Lag times increased by 55.9% in the impacted site and declined by 17.5% in the control catchment. Flow duration curve analysis showed a larger reduction in frequency of high flows, following beaver dam construction, with declines of Q5 exceedance levels of 33% for the impacted catchment and 15% for the control catchment. Using event total rainfall to predict peak flow, five generalized linear models were fitted to test the hypothesis that beaver dams attenuate flow, to a greater degree, with larger storm magnitude. The best performing model showed, with high confidence, that beaver dams attenuated peak flows, with increasing magnitude, up to between 0.5 and 2.5 m^3^ s^−1^ for the 94th percentile of event total rainfall; but attenuation beyond the 97th percentile cannot be confidently detected. Increasing flow attenuation, with event magnitude, is attributed to transient floodplain storage in low gradient/profile floodplain valleys that results from an increase in active area of the floodplain. These findings support the assertion that beaver dams attenuate flows. However, with long‐term datasets of extreme hydrological events lacking, it is challenging to predict the effect of beaver dams during extreme events with high precision. Beaver dams will have spatially variable impacts on hydrological processes, requiring further investigation to quantify responses to dams across differing landscapes and scales.

## Graphical abstract

Beaver dams are known to alter hydrological regime. We analysed 1246 hydrological events from 7 years before and 3 years after the construction of a beaver dam sequence, using a Before After Control Impact experimental design, showing that, following beaver dam construction: (i) hydrograph geometry was attenuated with longer lag times and reduced rising limb gradient, and (ii) flow attenuation increased with storm magnitude. The floodplain processes that contribute to flow attenuation are discussed.

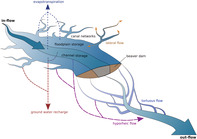

## 1 INTRODUCTION

Where beavers construct dams, there can be a transformative impact on the fluvial landscape. Beaver dams reduce stream longitudinal connectivity, simultaneously increasing lateral connectivity (Brazier, Puttock, et al., 2020; Puttock et al., 2021). This often results in the reinstatement of channel‐floodplain interactions, enhancing hydrological connectivity and driving the creation of dynamic, structurally complex wetlands (Brazier, Puttock, et al., 2020; Gurnell, 1998; Larsen et al., 2021). The enhancement of biodiversity, through the re‐establishment of such wetland environments, is well known (Law et al., 2016; Stringer & Gaywood, 2016). However, the coincidental impacts of beaver dams on hydrology are less well understood from a process‐based perspective.

Beavers construct dams to increase local water depth so that they can: (i) enhance security from predation by raising water levels above burrow/lodge entrances (Gurnell, 1998); (ii) improve access to foraging resources and reduce terrestrial movement, which is higher risk and greater effort (Campbell‐Palmer et al., 2015); (iii) to prevent freezing of water during the winter; and (iv) to store food resources, in the form of a woody cache, beneath the water surface (Campbell‐Palmer et al., 2015). Typically, dams are constructed in small rivers <6 m wide and <0.7 m deep (Hartman & Tornlov, 2006). Other factors that influence dam construction and density are building material availability, stream power, stream gradient and stream width (Dittbrenner et al., 2018; Graham et al., 2020; Macfarlane et al., 2017). It is typical for dams to be constructed in low‐medium gradient headwater streams ≤5th order (Graham et al., 2020; Gurnell, 1998; Rosell et al., 2005; Stevens et al., 2007). In larger rivers, where water is suitably deep, beavers are unlikely to build dams in the main channel (Brazier, Puttock, et al., 2020; Gurnell, 1998; Hartman & Tornlov, 2006).

Dams exert strong controls over hydrological processes during both high and low flows. The retention of more water within catchments has been shown to maintain base flows mediating the impacts of drought conditions (Majerova et al., 2015; Nyssen et al., 2011; Smith et al., 2020). It has also been shown that, in small headwater streams, beaver dam complexes can contribute to the attenuation of peak flows during hydrological events (Nyssen et al., 2011; Puttock et al., 2021, 2017), reduce mean flow velocity (Green & Westbrook, 2009) and provide significant stormflow storage which increases water‐residence times (Grygoruk & Nowak, 2014; Gurnell, 1998; Westbrook et al., 2020; Woo & Waddington, 1990). This flow attenuation effect has been attributed to the following causes:

- Beaver ponds provide a reservoir in which stormflow can be temporarily or transiently (Westbrook et al., 2020) stored before being released more slowly from the pond than it entered. The contribution of this mechanism is debated (Devito & Dillon, 1993; Larsen et al., 2021; Westbrook et al., 2020) because the available storage/freeboard behind dams is typically very small (Larsen et al., 2021) as pond depth is normally controlled by dam crest height; The effect of this attenuation mechanism is likely to be largely controlled by dam structure and flow state (Ronnquist & Westbrook, 2021; Woo & Waddington, 1990)
- Beaver dams, their wetlands and associated canals increase hydraulic roughness slowing flow and increasing water depth. Though these functional processes have been discussed (Grudzinski et al., 2020; Gurnell, 1998; Puttock et al., 2017; Puttock et al., 2021; Westbrook et al., 2020), their relative contribution is not known and is most likely variable (Larsen et al., 2021).
- Beaver dams enhance floodplain activation; water is therefore more readily stored within and on the floodplain. This effect was documented by Westbrook et al. (2006, 2020) and modelled by Neumayer et al. (2020), who predicted attenuation only in a stream situated in an unconfined low‐profile floodplain valley (Nanson & Croke, 1992).
- Some attenuation may also occur through groundwater and evapotranspirative losses (Larsen et al., 2021; Westbrook et al., 2006). Whilst the contribution of these mechanisms has not been quantified in beaver wetlands, it is likely that, following dry antecedent conditions or in arid locations, the effect of groundwater flow could exceed that of evapotranspiration.

With the assumption that transient storm water storage is limited to beaver ponds alone, like a flood storage basin, it can be assumed that a beaver pond sequence has a finite storage volume which will be reached quite rapidly during a storm event. A greater volume of storage may be available where the freeboard behind dams is large (Larsen et al., 2021) and or dam flow state is classified as underflow; that is, where water flows through holes in the dam structure below the of dam crest; Ronnquist and Westbrook (2021). Such observations have also been described for man‐made leaky wooden dams which, in many respects, aim to mimic the natural processes created by beaver dams; Norbury et al. (2021) note the importance of porosity in man‐made structures for regulating peak flows. Several studies have now reported increasing attenuation with higher flows (Nyssen et al., 2011; Puttock et al., 2021; Westbrook et al., 2020) which cannot be explained by pond storage alone. A key factor that may help to explain these processes is the structural heterogeneity of beaver wetlands which is not limited to the dam structures themselves but includes the often dense and expansive canal networks that beavers create across floodplains (Grudzinski et al., 2020). This structural complexity likely plays a crucial role in controlling numerous hydrological and floodplain processes. This study aims to explore further the dynamics of flow attenuation and discuss what mechanisms might be driving these observed increases in attenuation.

Land use intensification and channel modification are responsible for widespread fluviogeomorphic degradation such as channel incision and floodplain disconnection (Brown et al., 2018; Kondolf, 1997). In combination with projected intensification of storm events, flooding is likely to become more acute (O'Briain, 2019). Restoring natural processes and promoting water retention in catchments may help to ameliorate such impacts (Ellis et al., 2021).

This research analyses the mechanisms by which beaver dams impact stream hydrology using a Before After Control Impact (BACI) experimental design (Bilotta et al., 2016; Smith, 2014). A companion piece of analysis is also published as part of a multi‐site comparison in Puttock et al. (2021) that demonstrated flow attenuation across sites. Herein, focusing on one site in detail, we expand on these findings to consider the causes of observed changes to hydrological regime.

Puttock et al. (2021) demonstrate the effect of flow attenuation across multiple sites containing beaver dams. This was done using additive regression models comparing total event rainfall and peak event discharge with beaver presence as an additive covariate. In this context, a simple, parsimonious model enabled clear comparison between geographically disparate locations. These models assume an equal magnitude of attenuation with increasing rainfall intensity. This assumption may be appropriate if beaver dams behave as storage ponds; but, as mentioned above, given the small freeboard upstream of many dams, this mechanism cannot explain the increased attenuation for subsets of larger magnitude events observed by Puttock et al. (2021). This suggests that there is an interaction effect between total event rainfall and beaver dam presence, that is, with increasing storm magnitude, more attenuation occurs, and the slopes of the regression diverge for events before/after beaver. Therefore, an alternative or additional mechanism of flow attenuation to pond storage is required to explain this phenomenon.

This study tests the following hypotheses to advance our process‐based understanding of the functional impact that structural change, brought about by beaver dams, delivers during hydrological storm events.

*Flow entering the beaver dam complex is slowed, resulting in increased lag times between peak rainfall to peak flow*.

*Storm event peak flows are lower following the construction of a beaver dam sequence and the amount of attenuation increases with total event rainfall*.

Based on the outcomes of hypotheses one and two, a conceptual model is proposed describing the mechanisms of flow attenuation at the beaver dam complex and consider how this may form the basis of future work across a wide range of landscape types.

## 2 METHODS

### 2.1 Site descriptions

**FIGURE 1:**
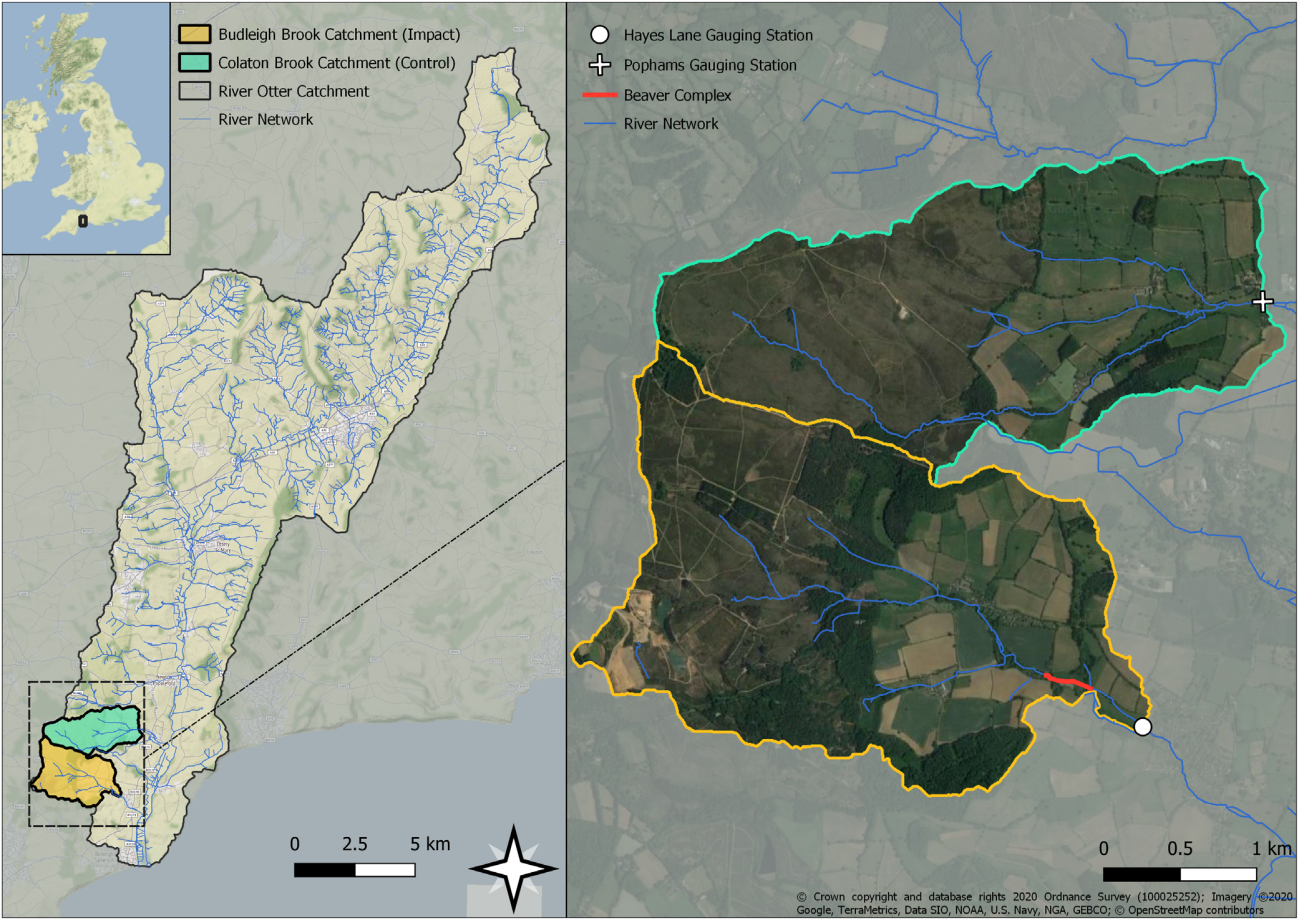
Left—Budleigh Brook (yellow) and Colaton Brook (green) catchment locations; right—location of EA gauging stations for the two catchments and the beaver complex on Budleigh Brook. Budleigh Brook has a catchment area of 6.3 km^2^ comprising managed grassland, outdoor pig farming, arable farming, heath, and woodland. The Colaton Brook catchment has an area of 5.5 km^2^ with dominant land uses including: Heathland, managed grassland, arable farming, and woodland

Two catchments are considered in this study; Budleigh Brook, the impacted site that contained a beaver complex and Colaton Brook, the control site, which had no evidence of beaver activity throughout the entire monitoring period (Figure 1).

#### 2.1.1 Budleigh Brook

Beavers have been active in the Budleigh Brook catchment since January 2017. The catchment is located within the wider River Otter Catchment (Brazier, Elliott, et al., 2020) and was colonized naturally (as opposed to being the site of beaver release). The precise time of colonization is unknown, though beaver signs, including damming, were observed on February 1st, 2017, therefore the period August 2016 to January 2017 (possible time of colonization) was excluded from this study.

Approximately 1 km of 3rd order channel is contained within the occupied beaver territory (ca. 3 ha); up to six dams had been constructed, within this reach, during the monitoring period. The contributing catchment area is 6.3 km^2^ and has mixed land use comprising: managed grassland, outdoor pig farming, arable farming, heath, and woodland. Climatic conditions at Budleigh Brook are temperate, with a mean annual maximum temperature of 12.6°C and a mean of 1065 mm of rainfall annually (Met Office, 2020).

**FIGURE 2:**
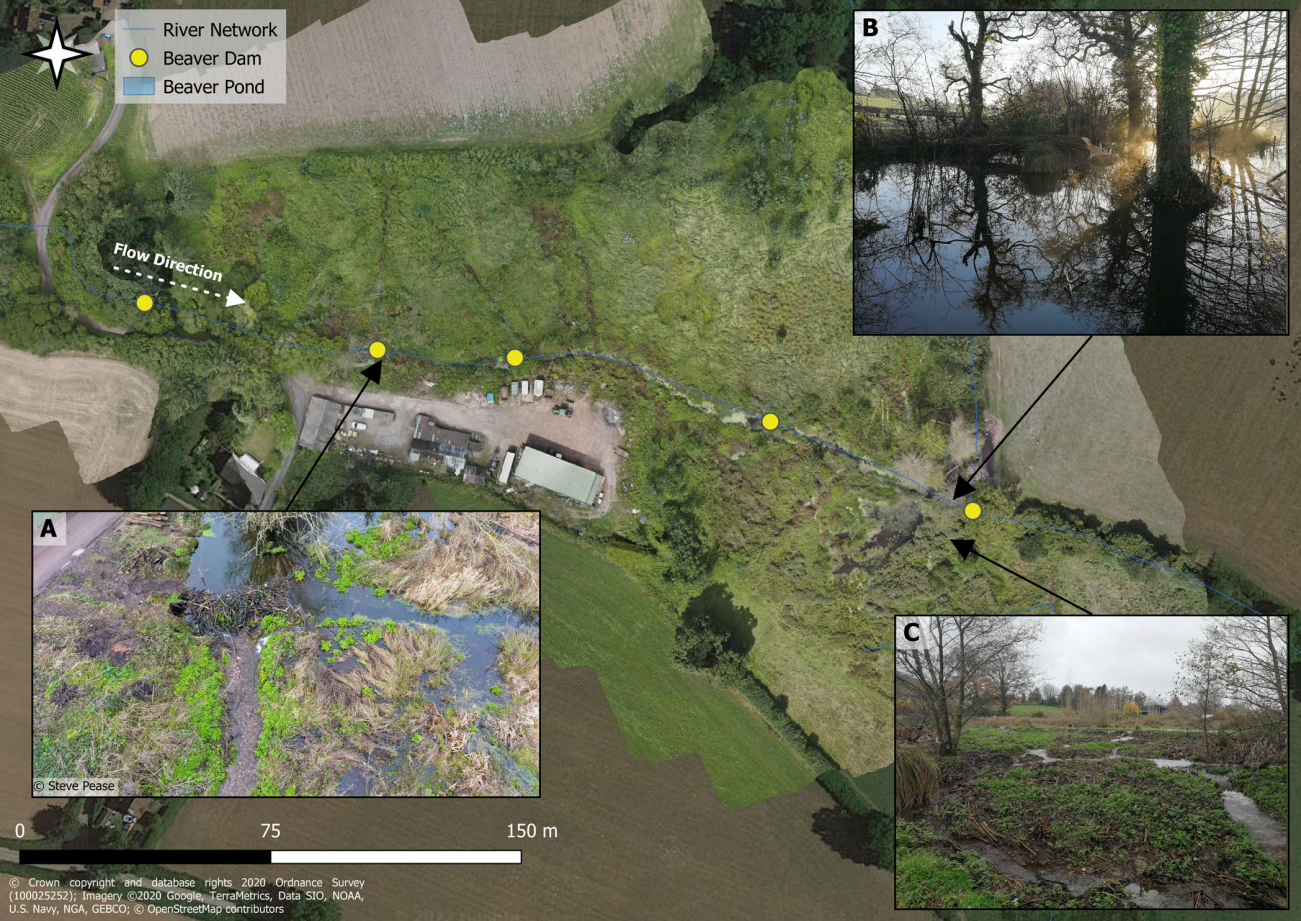
Budleigh Brook beaver dam complex showing dam and pond locations. (a) Diversion of water out of bank into the floodplain; (b) the main lodge pond; (c) development of multithread complex channel planform. The base map shows a drone‐derived orthomosaic of the site overlain on Google Earth Imagery

Beavers have significantly modified the site via the construction of dams as shown in Figure 2. The first and largest dam is located at the downstream end of the complex. This dam extends ca. 75 m across the floodplain and has caused the formation of numerous flow paths through the floodplain downstream of the structure. The pond, generated by this dam, has a surface area of ca. 1900 m^2^ and contains the beaver lodge. Several other dams have since been constructed upstream; presumably to improve mobility for accessing alternative food resources. A second pond with an area of ca. 300 m^2^ has been constructed further upstream. Some of these dams have been managed (removed or height reduced) to prevent surface water flooding of a nearby road (Brazier, Elliott, et al., 2020). Several other small dams exist but are not large enough to form a floodplain pond, but still impound water within the channel and push water onto the floodplain at high flows. The largest dam was constructed before February 2017 and has remained relatively stable since; upstream dams have collapsed and been rebuilt multiple times throughout the study period.

The Hayes Lane gauging station is located ca. 700 m downstream from the beaver dam complex (lat., long.: 50.6561, −3.3249) with no other channels entering the stream between the beaver dams and the gauging station. This gauging station is owned and maintained by the Environment Agency. The gauge is set within a stilling pond upstream of a weir which comprises a double‐trapezoidal channel profile along its crest (SI 1). The gauging station was constructed to provide an early flood warning system to the residents of East Budleigh, a community with properties at risk of flooding (Brazier, Elliott, et al., 2020).

Further information about the site can be found in Brazier, Elliott, et al. (2020) and Puttock et al. (2021).

#### 2.1.2 Colaton Brook

The neighbouring catchment, to the north of Budleigh Brook is Colaton Brook (Figure 1). Colaton Brook is also a 3rd order stream with a contributing catchment area of 5.5 km^2^ upstream of the flow gauge. The land use includes heathland, managed grassland, arable farming, and woodland. Pophams gauging station (Lat., Long.: 50.68125, −3.314561), also owned and maintained by the EA, provides 15‐min interval flow measurements. Beavers were not resident in this catchment during the study period and no beaver signs have been located upstream of the gauging station (Brazier, Elliott, et al., 2020; Brazier, Puttock, et al., 2020; Graham et al., 2022). This, the comparable size, stream order, distribution of land use and proximal location of the Colaton Brook catchment make it a highly suitable control.

### 2.2 Data processing/cleaning

The Hayes Barton gauge on the Budleigh Brook (impact) has not been rated for discharge measurement by the Environment Agency and reports only depth. The gauging station measures depth at 15‐min intervals and a record exists from July 2009 to present. Flow was estimated from this depth record using the following procedure. An area‐velocity flow meter (NivuFlow Mobile 750, Nivus, Germany) was installed for 2 months (December 2019–February 2020), 50 m upstream of the gauge/weir crest, within a stable, uniform trapezoidal channel (SI 1). Depth at zero flow was calculated by surveying the depth of flow over the weir crest and subtracting this from the gauged depth. A flow‐depth rating equation (SI 2) between measured flow and depth at the gauging station was generated using piecewise spline regression, as described in Fenton (2018), using the splines package (R Core Team, 2020). The depth at zero was used to anchor the rating curve through zero at this point. The rating equation was then applied to the full time series: from July 2009 to March 2020 (excluding the period August 2016 to January 2017 when the presence of beavers was uncertain).

Data from both the impact (Budleigh Brook) and control (Colaton Brook) sites were cleaned to remove visibly erroneous sections of the time series. These sections occurred during periods of maintenance where the stilling pond was drained. Further cleaning of the data was required to remove noise occurring at low flows. This step was necessary in advance of the automated event extraction to prevent the misidentification of events. An automated cleaning strategy was used: quantiles, for a specified time window (in this instance 12.5 h) at the 25th and 75th percentile, were calculated (termed *Q25*th and *Q75*th, respectively); a rolling quantile for the 70th percentile, for a one‐month period, was also calculated (*MQ70*). Where (*Q75*th − *Q25*th) > *MQ70*, the flow was considered elevated and any fluctuation in flow driven by precipitation; therefore, measured discharge was used. Where (*Q75*th − *Q25*th) *< MQ70*, the flow was considered to be low and not responding to a flow event; therefore a 7.5 h rolling mean for *Q* was used in place of measured *Q* to smooth out sensor noise occurring during low flows. The aim of this cleaning was to remove common noise associated with low flow measurement. No cleaning was therefore applied to flow event peaks, which was the dominant focus of this analysis.

### 2.3 Rainfall calculation

A rainfall record was required alongside the flow data to understand the precipitation volume and rate that contributes to each flow event. There were no historic rainfall gauges within either impact or control catchments that cover the full flow record used in this study. Further, rainfall is spatially variable and data from a single rain gauge can be problematic (Younger et al., 2009; Zeng et al., 2018). Therefore, rainfall radar data, derived from the NIMROD system (Met Office, 2003) were used. NIMROD data are provided as gridded total rainfall with spatial and temporal resolutions of 1 km and 5 min, respectively. Total rainfall for each time step was extracted for each site's contributing catchment area and converted to mean rainfall rate, before aggregating to 15‐min intervals to align with the temporal resolution of flow data, as per Puttock et al. (2021). Data download and conversion was conducted using Python (Python Software Foundation, 2019) and raster statistics were extracted with R (R Core Team, 2020) using the exactextractr package (Baston, 2020). The full hydrological time series is provided in SI 3.

### 2.4 Event extraction

The extraction of rainfall‐runoff events and corresponding metrics was undertaken using a semi‐automated rule‐based approach for the identification and pairing of rainfall and flow features from sub‐hourly observations (Puttock et al., 2021). Slow flow/fast flow was estimated by implementing flow separation on the padded time series (2880 reflected values, i.e., 2 days) with five passes of a single parameter recursive digital filter (alpha value of 0.98) after Ladson et al. (2013).

Rainfall events were classified as periods of rainfall over the median, categorized as either continuous (rainfall occurring on consecutive time steps) or isolated (SI 4). The span for minimum permitted gap between continuous periods was set through visual inspection and rainfall event periods separated by less than this span (90 min) were merged. To adapt thresholds for interannual variation and seasonality, while retaining a consistent approach, flow events were delineated using the digitally filtered fast flow (Ladson et al., 2013; Puttock et al., 2021). By default, the first timestamp in the event window was set to the start of the rainfall event ongoing at the beginning of the response event; or if no rainfall event was ongoing, the preceding rainfall event was used. Where a response event was paired with the same initiating rainfall as the previous event, it was assumed that contributing rainfall for the new event occurs during the falling limb, and the event window was bounded by the peak of the previous flow response.

All classified events were checked via visual inspection; erroneous/implausible events were removed from the analysis when, for example, the hydrograph geometry is angular and likely results from a sensor error or draining of the stilling well for maintenance; 91 and 13 events were removed for impact and control catchments, respectively. Event peak flow and total rainfall were calculated for each retained event window; with total event numbers of 612 and 634, for impact (Budleigh Brook) and control (Colaton Brook) catchments, respectively.

### 2.5 Data/statistical analysis

All statistical analysis and data visualization was undertaken using R (4.0.2) (R Core Team, 2020). the following packages were used: tidyverse (v1.3.1) (Wickham et al., 2019), lubridate (v1.8.0) (Grolemund & Wickham, 2011), stats (v4.1.0) (R Core Team, 2020), broom (v0.7.10) (Robinson et al., 2021), glm2 (v1.2.1) (Marschner, 2011), performance (v0.8.0) (Lüdecke et al., 2021), mgcv (v1.8.35) (Wood, 2017, 2011, 2004, 2003), gt (v0.3.1) (Iannone et al., 2020), gridExtra (v2.3) (Auguie, 2017), and ggpattern (v0.3.2.1) (FC & Davis, 2021).

To address H1, that the beaver dam complex slowed flow and increased lag times, a hydrograph averaging technique was adopted. Hydrograph data, including flow, rainfall and time were extracted for all events across both sites; events with a duration >95th percentile (36.75 h) were removed as these longer events were too few in number to model robustly with this approach. For each site, two general additive models (GAM) were fitted to compare both rainfall intensity and stream flow change with time since event start. Each GAM was fitted using the form below:

$$\text{Response}\sim s\left(\text{Time},\mathrm{by}=\text{Beaver Presence},k=i\right)+\text{BeaverPresence},$$

where *Response* is either rainfall rate or stream flow depending on which model is fit, *Time* is the time since event start and *Beaver Presence* is the presence/absence of beaver. The ***s*** function defines the smoother used within the GAM where *Beaver Presence* is included as a covariate, such that the smoother is fit independently for each factor level (i.e., for events where beavers were present and absent). The *k* argument is the basis dimension of the smoother term (approximating the degrees of freedom); values of 5 and 10 (the default, i.e., *k* − 1) were used for stream flow and rainfall models, respectively. A *k* value of 5 was required to reduce overfitting of the stream flow model. A cubic regression spline smoother was used for all models. The GAMs provide an approximation of the mean hydrological event response before and after beaver, providing important insight into changes in event geometry. However, as rainfall and flow are not explicitly used in the same model, comparison between the before/after beaver responses must be considered alongside changes in mean rainfall and the response at the control site. All GAMs were fitted using the mgcv package (Wood, 2017, 2011, 2004, 2003). Herein these models are referred to as GAM hydrographs. Average lag times before and after beaver were calculated by differencing the predicted peak times of rainfall and flow.

To evaluate the impact of beaver dams on overall hydrological regime, flow duration curves (FDC) (Vogel & Fennessey, 1995) were generated for both control and impacted sites. FDC metrics including R2FDC and Q5:Q95 ratio were also calculated to evaluate the changes to the FDC. R2FDC describes the slope and the variability of flow in the middle third of the FDC in logarithmic scale (Ochoa‐Tocachi et al., 2016); a value closer to zero therefore indicates increased hydrological stability in the central flow range. Q5:95 ratio is used as a flashiness index (Jordan et al., 2005) to describe the range in flow conditions; lower values indicate that a system is less flashy, having a slower response to rainfall.

Given that meteorological effects, precipitation in particular, normally exert strong control over the flow regime of temperate perennial river systems, it is important to consider this when investigating the effect of a non‐meteorological disturbance on a river system (such as a beaver dam). Therefore, following the methods in in Puttock et al. (2021), General Linear Models (GLM) were used to consider how peak flows were related to total event rainfall, with beaver presence included as an additive covariate and site as an interactive covariate (M1 in Table 1). Site was included as a model term to compare the difference between the control and impacted locations. We extend this analysis and compare this model alongside four alternative models as shown in Table 1. All models were fitted using a Gamma error distribution because small hydrological events were far more common than large events and therefore a Gaussian (normal) error distribution is not appropriate. Models were fitted with increasing complexity; M2 and M3 included beaver presence as an interactive covariate, enabling freedom in the regression slopes between the two factor levels; M2 and M3 were fitted using identity and log link functions, respectively. M4 and M5 use the second‐degree orthogonal polynomial of total event rainfall as the continuous control variable in addition to beaver presence and site as interactive covariates; M4 and M5 were fitted using identity and log link functions, respectively. Polynomial regression was adopted following the inspection of normalized residual plots for M1:M3 (SI 6) which indicated the potential existence of a nonlinear response due to a trend in residual plots. M1 and M2 were fitted using the glm2 package (Marschner, 2011); M3:M5 were fitted using the glm function from the stats package (R Core Team, 2020).

**TABLE 1:** A description of the fitted general linear models. The model number is the reference used in this paper, its form is presented as pseudo code in line with R syntax and the link function denotes the relationship between the linear predictor and the mean of the distribution. Models 1 and 2 were fitted using he glm2 package (Marschner, 2011) and Models 3:5 were fitted with the glm function from R's stats package (R Core Team, 2020)

*Model ID*	*Model form*	*Link function*
M1	EventMax.Flow~Total Rainfall+Beaver Presence×Site	*Identity*
M2	EventMax.Flow~Total Rainfall×Beaver Presence×Site	*Identity*
M3	EventMax.Flow~Total Rainfall×Beaver Presence×Site	*Log*
M4	$\text{Event}\;\mathrm{Max}.\;\text{Flow}\sim \text{Poly}\left(\text{Total Rainfall},2\right)\times \text{Beaver Presence}\times \text{Site}$	*Identity*
M5	$\text{Event}\;\mathrm{Max}.\;\text{Flow}\sim \text{Poly}\left(\text{Total Rainfall},2\right)\times \text{Beaver Presence}\times \text{Site}$	*Log*

Total event rainfall was chosen as the main control variable, rather than other rainfall metrics, as it was found to have a strong correlation with peak flow across the sites in Puttock et al. (2021) and therefore allowed for comparison with multiple other locations. The Pearson's r correlation values between peak flow and total event were 0.483 and 0.595 for impact and control catchments, respectively; similar values were given for mean rainfall and rainfall rate. The inclusion of site and its interaction with beaver presence/absence is crucial for determining whether any effect of beaver was coincidental or not. Where the interaction between beaver presence and site is significant, it can be deduced that the impact of beaver, at the impacted site, on peak flow is significantly different from the control site. This well‐established Before‐After‐Control‐Impact (BACI) design offers robust inference for systems where all influential variables cannot be known or measured (Bilotta et al., 2016; Smith, 2014).

Following the evaluation of model diagnostic plots (SI 6), produced using the performance R package (Lüdecke et al., 2021), Model performance metrics (Table 2) including Akaike information criterion (AIC), Bayesian information criterion (BIC), *R* ^2^ (Nagelkerke, 1991), Root Mean Square Error (RMSE) and Sigma, and qualitative plausibility, the M5 model (Polynomial model with log link) was selected as the best model. Though it did not produce the lowest AIC value or highest *R* ^2^ (second best of the tested models), it was found to most effectively capture the uncertainty in the data distribution; particularly so for large events with >40 mm total rainfall where only three events of this magnitude were captured post beaver. This was particularly evident in the residual diagnostics plots where there was lower deviance in residuals for larger predicted values indicating a greater reliability for predictions during larger storm events, which is of key interest herein. Using M5, the peak flow attenuation was calculated across the total rainfall range by calculating the difference between predictions with and without beaver. Attenuation was then plotted against the percentile total rainfall to understand how beaver dams attenuate flow across the observed event total rainfall range.

**TABLE 2:** Model performance metrics for the GLMs presented in Table 1 and Figure 5; used to help with model selection. *K* denotes the number of terms used for fitting the model, Akaike information criterion (AIC) and Bayesian information criterion (BIC) are a descriptor of model quality where a lower value indicates a better fit. *R* ^2^ was derived according to Nagelkerke (1991). Estimates of model precision are given by root mean square error (RMSE) and sigma

*Model ID*	*K*	*AIC*	*BIC*	*Nagelkerke's R* ^2^	*RMSE*	*Sigma*
*M1*	5	−12.35	18.41	0.64	1.24	0.97
*M2*	8	−146.49	−100.34	0.69	1.17	0.93
*M3*	8	−200.10	−153.95	0.70	1.77	0.91
*M4*	12	−244.04	−177.38	0.72	1.10	0.90
*M5*	12	−226.72	−160.06	0.72	1.09	0.90

## 3 RESULTS

### 3.1 (H1) Flow entering the beaver dam complex is slowed, resulting in increased lag times

**FIGURE 3:**
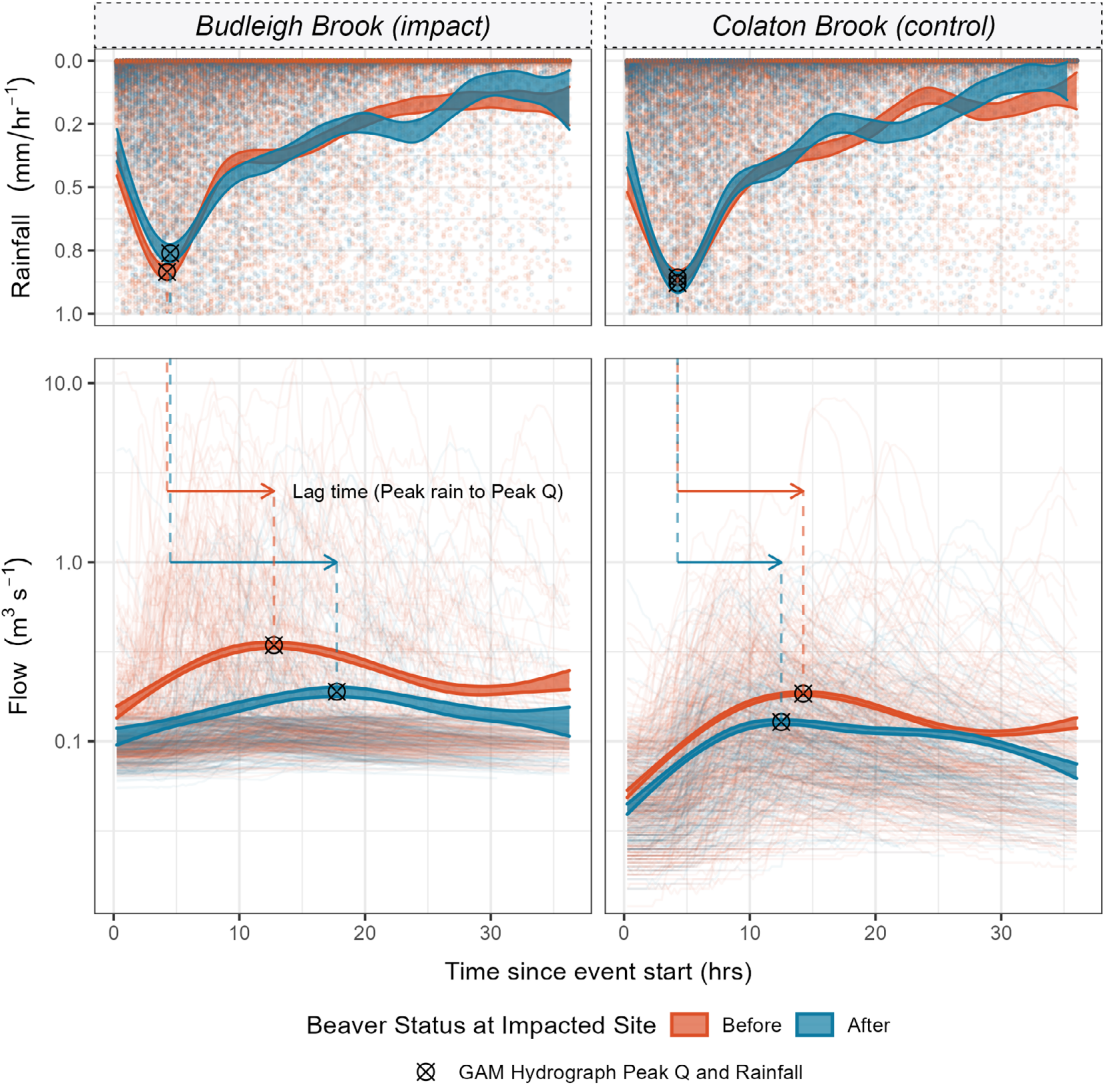
The 95% confidence limits of the general additive model (GAM) hydrographs are presented as the shaded ribbons; individual rainfall records are presented as points and individual event hydrographs are presented as lines. The plot enables semi‐quantitative assessment of changes in average hydrograph response following beaver dam complex construction. Average event peaks are shown as crosses; these demonstrate that an increase in lag times has occurred at the impacted site whilst a slight decrease in lag times occurred at the control

Figure 3 shows the GAM hydrographs; in the control site (Colaton Brook), a slight reduction in the mean peak flow and lag time is observed between the periods before and after beaver occupancy at the impact site. Differences in the shape of the GAM hydrograph before and after beaver are subtle indicating that, whilst mean event magnitude may have declined, the hydrological response to these events is relatively unchanged. In contrast, for the beaver‐impacted site (Budleigh Brook), there is a larger reduction in event magnitude but also a considerable deviation in hydrograph shape after the construction of the beaver dam complex. Most notably, the delayed event peak and reduced gradient of the rising limb; this increase in lag times (55.9%), in contrast to the decrease (17.5%) at the control site, is a strong indication of flow attenuation. GAM hydrograph slope gradient, during the lag‐time period, decreased at both sites by 67% CI [64, 72] and 29% CI [26, 33] for the impacted and control sites, respectively. Mean event rainfall intensity at the control site remained similar after beaver dam construction. This is the case too for the impacted site, although a slight decrease in peak rainfall following beaver colonization may explain, to some extent, the larger peak flow reduction observed at Budleigh Brook.

### 3.2 (H2) Storm event peak flows are lower following the construction of a beaver dam sequence and the amount of attenuation increases with larger rain events

**FIGURE 4:**
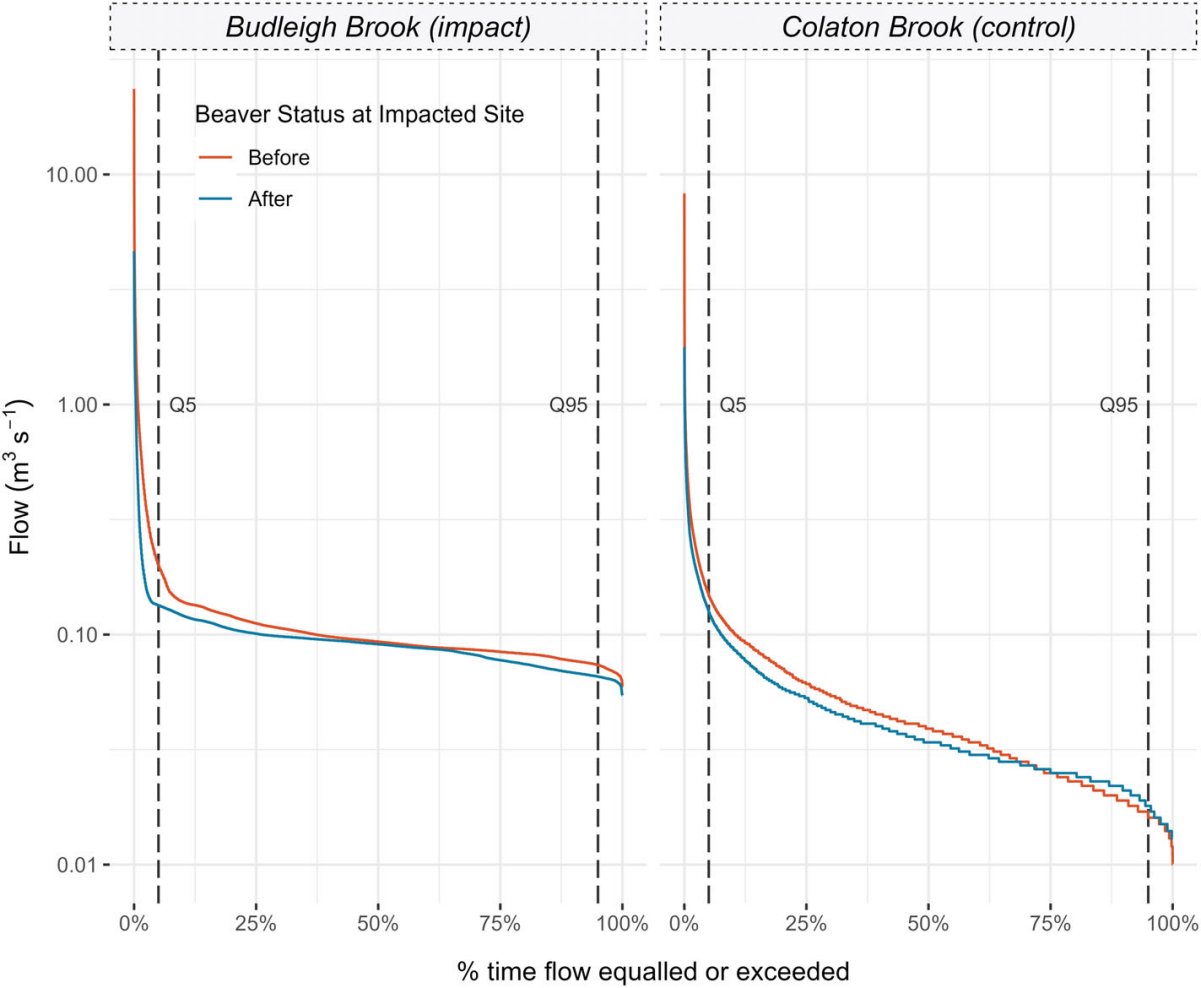
Flow duration curves (FDC) for Budleigh and Colaton Brook. These plots represent the proportion of time that a given flow is equalled or exceeded. Q5 and Q95 lines indicate the proportion of time the flow was greater or equal to the 95th and 5th flow percentiles, respectively

The FDC curves (Figure 4) and metrics (Table 3) clearly show that, at the impacted site, higher flows were less frequently observed; highlighted by a 33% decrease in the Q5 exceedance value at the impacted site and only a 15% reduction at the control. The results for low flows, at the Q95 exceedance limit, also differ; in the control catchment, an increase in Q95 of 12.5% was observed in contrast to the impacted site which experienced a 10% decline in Q95, following beaver colonization.

**TABLE 3:** Flow duration curve (FDC) metrics for the two sites, including: Mean and median flow; R2FDC which describes the slope and the variability of flow in the middle third of the FDC in a logarithmic scale (Ochoa‐Tocachi et al., 2016); Q5 and Q95 exceedance limits, and the Q5:Q95 ratio which is used as a descriptor of system flashiness

	*Mean*	*Median*	*r2fdc*	*q5*	*q95*	*Q5:Q95 ratio*
*Budleigh Brook* (*impact*)						
No Beaver	0.13	0.09	−0.23	0.20	0.07	2.72
Beaver	0.10	0.09	−0.20	0.13	0.07	2.04
% Change	−21.58	−2.49	−14.40	−33.29	−10.86	−25.16
*Colaton Brook* (*control*)						
No Beaver	0.06	0.04	−0.72	0.15	0.02	9.38
Beaver	0.05	0.03	−0.56	0.13	0.02	7.06
% Change	−15.18	−12.82	−21.25	−15.33	12.50	−24.74

Changes to the Q5:Q95 ratio flashiness index were comparable across both sites. R2DFC decreased in both sites with a 21% and 14% decrease in the control and impacted sites, respectively. Larger differences in Q5 and Q95 values, alongside a smaller reduction in R2DFC, at the impacted site indicate that the effect of the beaver dam sequence was particularly evident during the hydrological extremes, and less change was observed for intermediate flows, relative to the control site, which experienced larger changes in the central region of the FDC.

**FIGURE 5:**
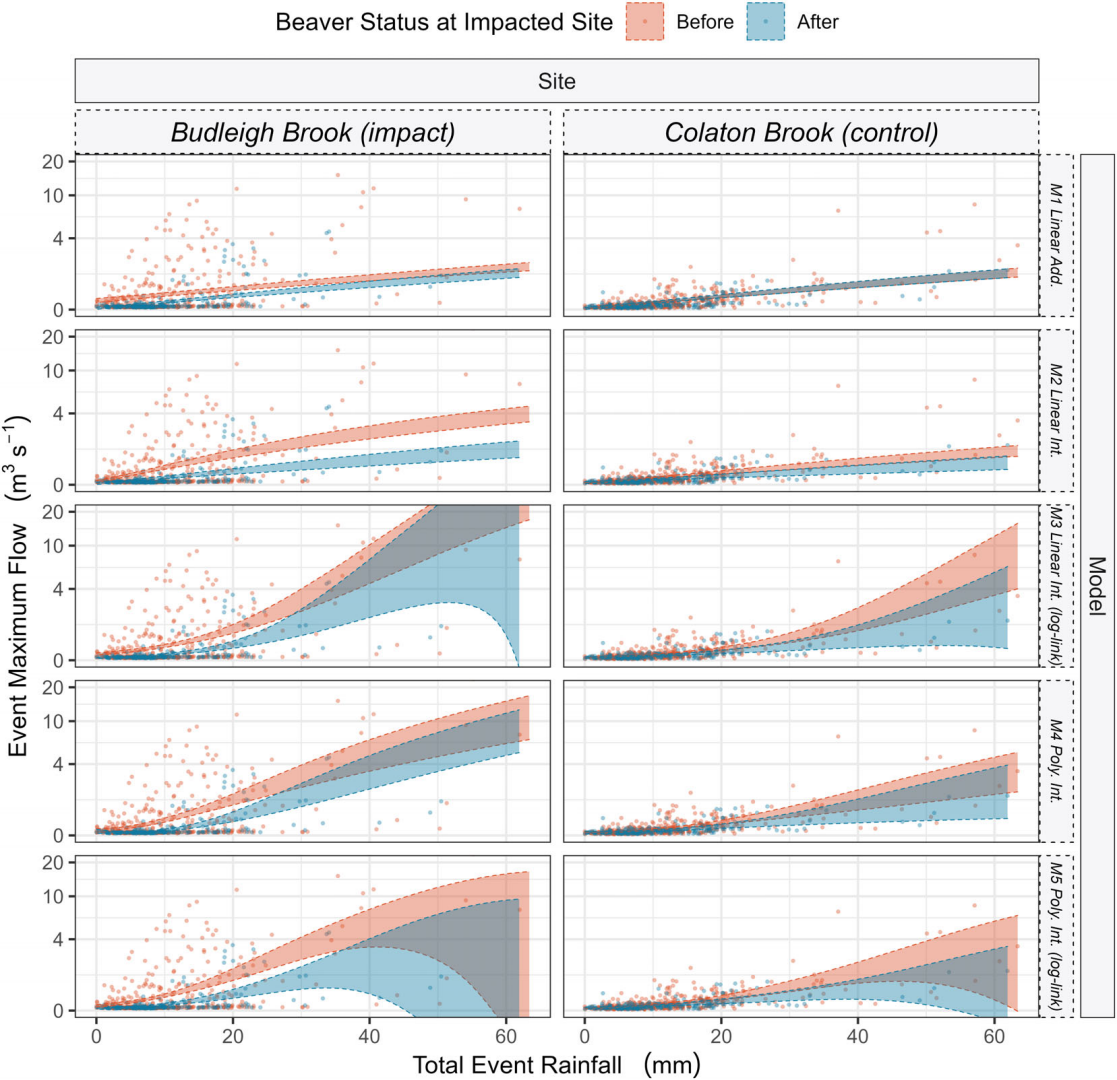
The general linear models fitted to determine the impact of a beaver dam sequence on the relationship between total hydrological event rainfall and peak event discharge. Shaded areas represent the 95% confidence intervals of the fitted models. Model IDs correspond to Table 1. *Add*. Indicates that beaver presence is included as an additive term, *Int*. indicates that it was included as an interactive effect. Where log‐link is not specified, an identity link was used. All models show that peak flows, for a given total event rainfall, decreased following beaver presence; for models M3:M5 there may only be confidence in this effect up to a given limit (where confidence intervals intersect)

**FIGURE 6:**
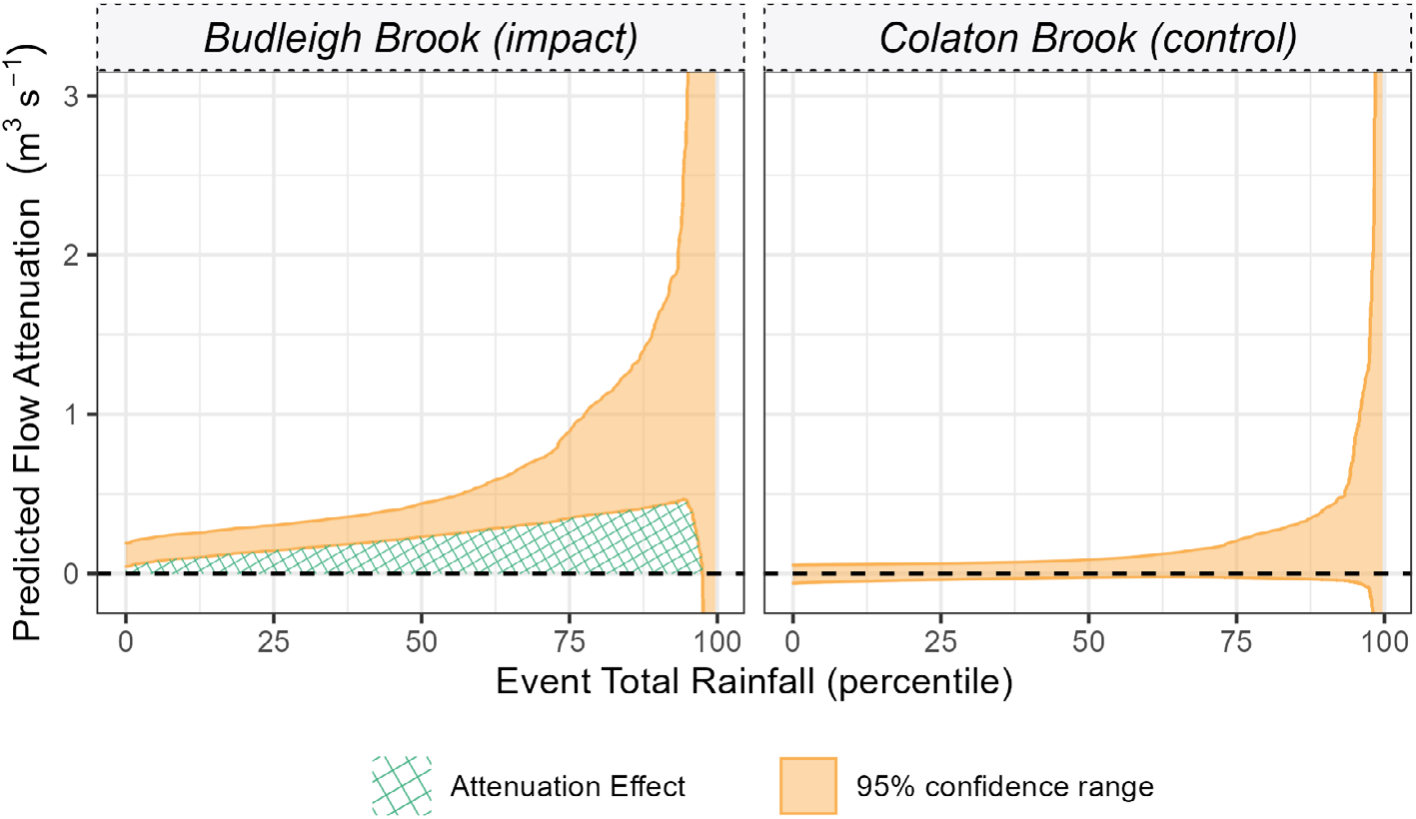
The predicted flow attenuation derived from the polynomial regression with log link (M5: Table 1, Figure 5). This is the difference between predicted flows pre and post beaver. Orange shaded regions describe the 95% confidence limits of the model. Where confidence limits are above the zero (dashed) line, there can be >95% confidence in attenuation—this area is shown by the green crosshatched area. Where the zero‐line falls within the confidence limits there is low confidence of observing flow attenuation

Models M1, M4 and M5 (Table 1 and Figure 5) found a significant (*p* < 0.05) interaction between beaver presence and site, indicating that there was a significant difference between the peak flows before and after beaver at the impacted site. Models M2 and M3 showed a significant (*p* < 0.05) interaction between beaver presence, site and event total rainfall which indicated that the slope of the relationship between total rainfall and peak flow is significantly different after beaver occupancy at the impacted site. Model summary tables are presented in SI 7. Raw data, and data density distributions for peak flows across each site are presented in Figure 6.

Nagelkerke's *R* ^2^ (Nagelkerke, 1991) values indicate that models M4 and M5 provide the best fit (Table 3). Although M4 has a better model fit than M5, based on AIC and BIC (Table 3), there is deviation in the residuals for the upper limits of fitted values (as shown in residual diagnostics plots given in SI 6 and fractionally lower RMSE (Table 3)); therefore, M5 was adopted, despite its marginally poorer fit, as we can have greater confidence in its inference for larger events.

**FIGURE 7:**
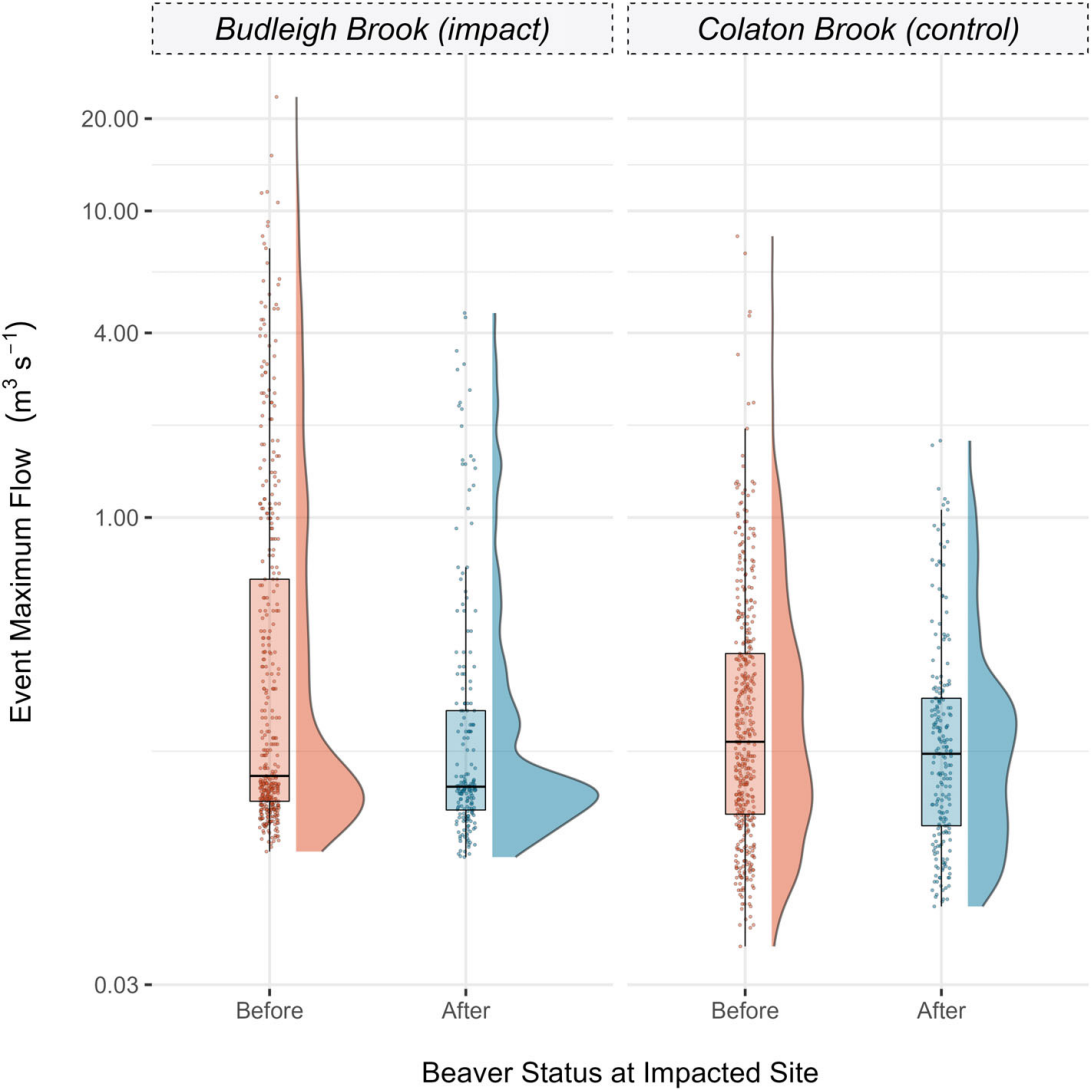
Raincloud plot showing the raw data, boxplot statistics and density distribution of peak flows for hydrological events in both Budleigh Brook and Colaton brook, before and after beaver the complex was established in the impacted site

Flow attenuation change with percentile total event rainfall is presented in Figure 7. For much of the total rainfall distribution, it was observed that 95% confidence intervals of the model (M5) were greater than zero; this region is highlighted in Figure 7 as a green hatched region. Peak flow attenuation was found to increase up to the 94th percentile with a magnitude of between 0.5 and 2.5 m^3^ s^−1^ (95% CI) equivalent to a peak flow reduction of between 23.4 and 76.5%. Beyond the 97th percentile, attenuation was estimated to be between 0 and 5.2 m^3^ s^−1^ and therefore could not be identified with confidence. No such attenuation was observed for Colaton Brook.

## 4 DISCUSSION

### 4.1 (H1) Flow entering the beaver dam complex is slowed, resulting in increased lag times

Increases in lag times and reduced rising limb slopes were three and two times greater, respectively, at the impacted site following beaver dam complex construction, than the control site (Figure 4). Given the significant interaction term between beaver presence and site in the selected GLM model (M5), there can be confidence that this change resulted from the creation of the beaver dam complex. The potential for beavers to increase lag times has been both observed (Burns & McDonnell, 1998; Larsen et al., 2021; Nyssen et al., 2011; Puttock et al., 2021, 2017; Westbrook et al., 2020) and modelled (Neumayer et al., 2020; Stout et al., 2017) in other studies. This flattening of the curve is a clear demonstration of the theory underpinning natural flood management (NFM) interventions, where the desired goal is to mediate the flow response, extending the duration of events such that the peak flow is reduced (Ellis et al., 2021; Lane, 2017; Norbury et al., 2021).

Key reasons for this attenuated hydrograph geometry are likely to include, the increased effective storage capacity (Gurnell, 1998; Larsen et al., 2021; Puttock et al., 2021, 2017; Westbrook et al., 2006; Woo & Waddington, 1990) and reduced flow velocities (Butler & Malanson, 1995; Green & Westbrook, 2009; Parker et al., 1985), driven by an increase in roughness (Larsen et al., 2021; Puttock et al., 2017).

### 4.2 (H2) Storm event peak flows are lower following the construction of a beaver dam sequence and the amount of attenuation increases with larger rain events

Attenuation estimates presented herein for Budleigh Brook and in Puttock et al. (2021), across three other sites in England, illustrated an attenuation effect for larger storm events. Increased attenuation with larger events was also reported by Nyssen et al. (2011), for a dam complex in Belgium, where a dam sequence was found to lower peak flows and increase flood flow return intervals. Westbrook et al. (2020) demonstrate that flood attenuation still manifests for even the largest of hydrological events; the authors found that, in Alberta during largest recorded flood in the Canadian Rocky Mountains, the majority (68%) of dams within the research area were resilient to high flows providing important storm‐water storage and increased water retention times both in ponds and laterally across adjacent floodplains.

The location considered herein and those others in (Puttock et al., 2021), sit within unconfined, low‐profile flood plain valleys. Modelling by Neumayer et al. (2020) demonstrated increased lag times due to beaver dams but only in valleys with wide and low gradient floodplain profiles. Local topography and channel/floodplain geomorphology are therefore likely to exert a strong control on attenuation processes (Brazier, Puttock, et al., 2020; Larsen et al., 2021; Westbrook et al., 2006). Available storage within ponds is also very likely to affect flow attenuation (Westbrook et al., 2020); however, it is highly variable. Where ponds are less full, prior to an event, they have greater capacity to store/attenuate flow due to the available freeboard (Larsen et al., 2021; Ronnquist & Westbrook, 2021; Westbrook et al., 2020). This mechanism will largely be controlled by the flow state of dams within a complex (Ronnquist & Westbrook, 2021; Woo & Waddington, 1990). As ponds fill during an event, water is stored in the pond during the transition between underflow/gap flow to overflow (i.e., overtopping) (Butler & Malanson, 1995; Devito & Dillon, 1993; Ronnquist & Westbrook, 2021). In addition to dam structure, antecedent conditions will also play a critical role in controlling pre‐event pond levels/flow state (Neumayer et al., 2020; Puttock et al., 2021, 2017). However, the largest dam in the Budleigh Brook pond complex is typically in a full or near to overflow state (Ronnquist & Westbrook, 2021), even after dry weather, and therefore the available freeboard, that is, pond storage is rarely more than 5 cm. This would approximate to ca. 95 m^3^ of available transient storage across the 1900 m^2^ pond. Though not insignificant, this volume cannot explain the continued increases in attenuation that were observed as this storage capacity would be rapidly filled during a large event. Therefore, additional attenuation mechanisms must have taken place.

### 4.3 Conceptual model of flow attenuation

**FIGURE 8:**
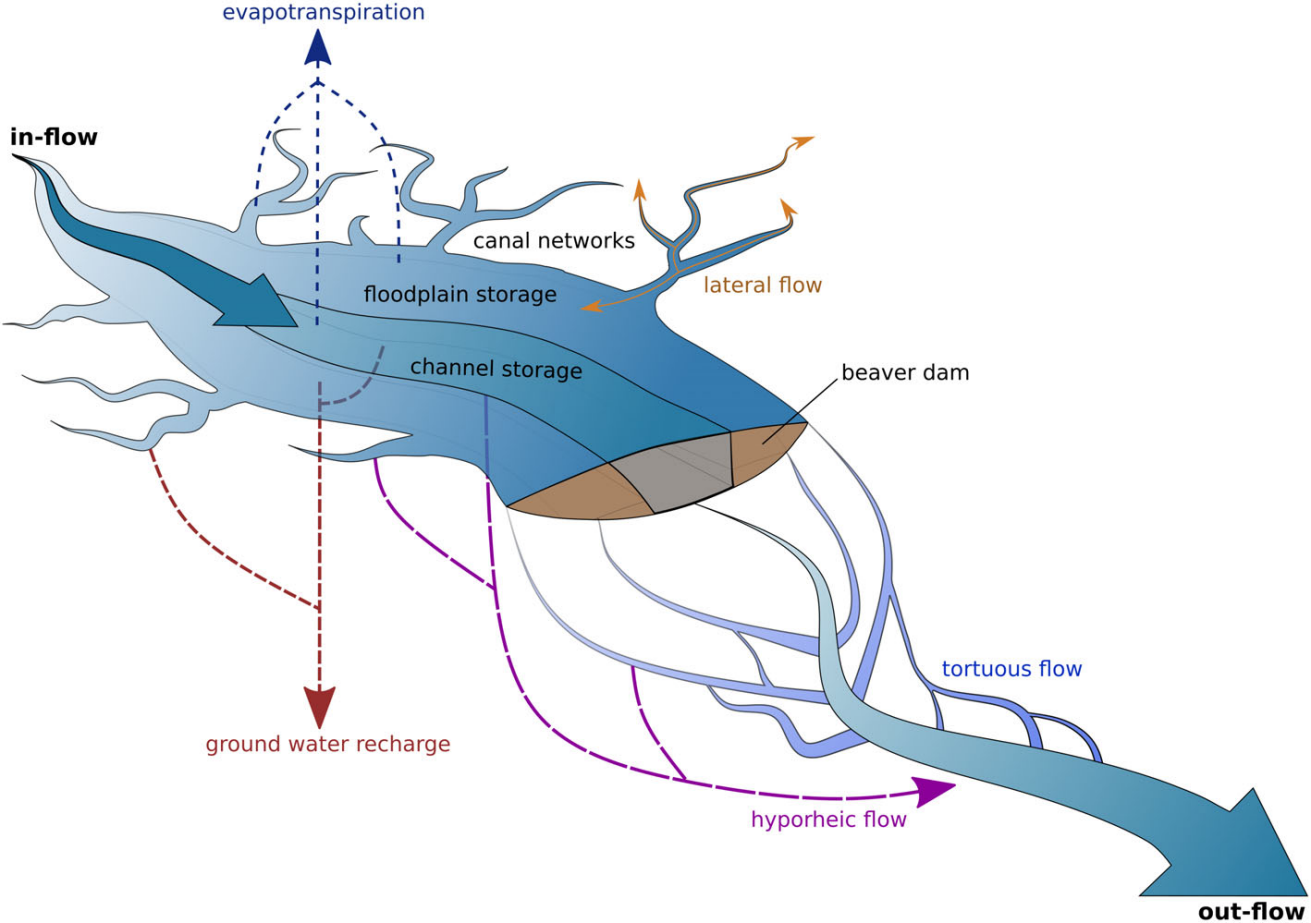
Conceptual model describing the mechanisms of flow attenuation within a beaver wetland with an unconfined floodplain. As flow and therefore the depth of water behind the dam increases, the area of activated floodplain also increases; this results in the formation of new flow, infiltration and evapotranspiration pathways. These pathways become longer and more tortuous as the flooded area expands during a flow event. Canals likely play an important role in transporting water laterally into the floodplain further enhancing floodplain connection

We suggest that, in a low‐profile floodplain valley, not confined by steep valley sides or impeded by man‐made features such as levées (Nanson & Croke, 1992), beaver dams will readily reconnect channel‐floodplain flow pathways, forcing water horizontally onto the floodplain as flows, and therefore pond levels behind the dam, increase. With increasing flow, water is diverted around/over/through the dam structure with ever increasing flow pathway length, tortuosity, roughness and depth. Simply put, beaver dams and their associated wetlands increase the active area of floodplain and therefore the surface area over which floodplain process can occur. This was observed by Westbrook et al. (2006) who documented large flow diversions extending up to 930 m downstream of the beaver dams. To reach a floodplain flow depth equivalent to the post beaver inundated extent, a flood with a >200‐year return interval would have been required. A conceptual diagram of these processes is presented in Figure 8 which highlights the multitude of hydrological pathways that are activated in a beaver wetland, when the channel and floodplain are reconnected by damming; these include surface processes but also subsurface flow both into the shallow hyporheic zone, and deeper aquifer storage, depending on the local soil and geological properties. Beaver canals further enhance these processes by diverting water to more distant regions of the floodplain yet again increasing the active floodplain area (Grudzinski et al., 2020); canals will provide both new flow pathways but also act as temporary storage areas during storm events.

The diverted flow increases water storage, but it is transient and dynamic; as noted by Westbrook et al. (2020) who found that the flood extent, during a very large flow event, was up to 20 m from the pond edge. This supports the idea that wider dams, that enhance floodplain connection, may exert a greater effect on peak flow due to the increased availability of floodplain storage, in addition to pond storage (Puttock et al., 2021). At some threshold discharge, the attenuation effect must plateau (though it is noted here that long, tortuous and high roughness flow paths will persist); this threshold approximates the point where floodplain inundation before beaver is equal to the inundation extent post beaver. In a confined valley, the area over which new flow pathways can form and water can be transiently slowed, stored and infiltrated/evaporated is substantially reduced and therefore this threshold/plateau will be reached more rapidly. Consequently, it is likely that beaver dams have the strongest effect in reducing peak flows in low‐profile valleys (Neumayer et al., 2020), where the structures are more likely to persist (Graham et al., 2020; Green & Westbrook, 2009; Macfarlane et al., 2017; Westbrook et al., 2020) and they can activate the floodplain rapidly and over larger areas enhancing transient storage and reducing overland velocities. The effect of beaver dams on high flows, therefore, varies spatially in response to topography, geomorphology, dam structure and dam density (Gurnell, 1998; Puttock et al., 2021; Ronnquist & Westbrook, 2021; Westbrook et al., 2020) but also temporally in response to antecedent climatic conditions. Further investigation in the hydrological effects of beaver dams in steeper river systems would be valuable though to understand the importance of lateral subsurface infiltration and its potential contribution to attenuating peak flows; such processes are likely to be highly dependent on local soil types.

These mechanisms of flow attenuation have important ramifications for where beaver dams may be desirable from a flood mitigation perspective and where projects replicating beaver dam processes, such as the use of beaver dam analogues (Bouwes et al., 2016; Munir & Westbrook, 2021), should be placed in order to yield flow attenuation benefits. These factors will need to be considered, alongside other potential impacts of dams, on biodiversity and economically valuable land/infrastructure. Beavers preferentially dam streams with wider floodplain extents (Dittbrenner et al., 2018) and with lower stream gradients (Dittbrenner et al., 2018; Graham et al., 2020; Hartman & Tornlov, 2006; Macfarlane et al., 2017) where available, often at locations immediately downstream of tributary confluences (Baskin et al., 2011). Therefore, it is possible that the greatest attenuation benefit will accrue during the initial stage of beaver population expansion as these preferred locations are occupied.

As beaver populations expand, family groups will abandon territories, leaving dams unmaintained. Where these dams have previously persisted for some time, they are often stabilized by vegetation (Johnson‐Bice et al., 2022; Pollock et al., 2014). Many of these dams can therefore remain in the landscape and continue to exert strong controls over surface water storage/flow routing at catchment and regional scales after the abandonment of territories (Johnson‐Bice et al., 2022). Abandoned dams may also influence storm event dynamics; so their impact on peak flow attenuation, and indeed low flow conditions, warrants further investigation.

The anthropogenic modifications of rivers systems globally, primarily through the intensification of land use, combined with dredging, drainage and the construction of permanent barriers like weirs/dams, that starve rivers of coarse sediment, have led to the widespread incision of river channels within their floodplains (Brown et al., 2018; Kondolf, 1997). For a floodplain to be activated, in an incised channel, a far greater flow is required (Pollock et al., 2014). It is likely therefore that the attenuation effect of beaver dams will be most substantive in these modified systems, where the ratio between the flow required for floodplain activation, pre/post beaver dam construction, is greatest. Where incision is not an issue and floodplains are (still) activated readily, the potential increase in attenuation, due to beaver may well be less pronounced as the river's hydrological response is likely to already be more natural and attenuated. This could explain why Burns and McDonnell (1998), who monitored the impacts of a large beaver dam complex and associated wetland on the hydrological regime of a forested catchment in New York State, observed flow attenuation but only to a very limited extent for large events.

### 4.4 Low flow considerations

Several studies report the amelioration of drought conditions downstream of beaver dam complexes (Majerova et al., 2015; Nyssen et al., 2011; Smith et al., 2020). This effect manifests because the water stored in beaver ponds leaks slowly, maintaining an elevated base flow. In contrast, it is suggested that increased evapotranspiration rates can lead to a decline in base flow discharge (Correll et al., 2000; Larsen et al., 2021; Meentemeyer & Butler, 1999; Woo & Waddington, 1990). FDCs for the impacted site appear to show no maintenance of base flow with low flows (<Q95) decreasing following beaver dam complex construction. Streamflow losses may have therefore increased following beaver reintroduction. This may have resulted from either increased evapotranspiration in the beaver wetland (Burns & McDonnell, 1998; Fairfax & Small, 2018), and/or increased groundwater recharge, following increased water residence time (Westbrook et al., 2006). The spatial extent of this effect is unknown, though it is conceivable that, due to the porous pebble bed geology of both catchments (Sherrell, 1970), that local groundwater losses may enhance low flows further downstream. Spatial variability in hydrological responses to beaver dams, during high flows, is frequently discussed (Brazier, Puttock, et al., 2020; Larsen et al., 2021); spatial and temporal variability, in the response to low flows, may also be significant and warrants further investigation.

### 4.5 Implications for future work

This study demonstrates that there are likely to be multiple mechanisms by which beaver dams attenuate high flows, most likely occurring simultaneously during flood events. There is strong evidence to suggest that this attenuation will increase with greater flows. However, given the large uncertainty for predicted attenuation during the largest of events, further work is required to understand the potential impact of beaver dams on flow, both at the site and catchment scale (Brazier, Puttock, et al., 2020; Larsen et al., 2021). Hydraulic modelling, such as that demonstrated by Neumayer et al. (2020) and Stout et al. (2017) is a vital step in understanding the effect of beavers at these extreme flows. The representation of beaver dams in such models is complex and challenging though, currently requiring dam structures to be defined by the limitations of software. For example, Neumayer et al. (2020) represent the interstitial gaps in the dam as a set number of small pipes; this pragmatic simplification is understandable, but no doubt could be improved with further empirical observation. A stronger dialogue between such empirical data and model development would help to refine parameters such as hydraulic roughness across beaver wetlands, rates of dam under/through flow, and when floodplain activation occurs during large storm events. It is also crucial to capture the variability in dam structure and dimension as demonstrated by Ronnquist and Westbrook (2021) and Hafen et al. (2020)—these factors, in addition to variable dam densities, are likely to exert strong controls on flow attenuation (Beedle, 1991). Therefore, further hydraulic modelling is required, to build on the work of Neumayer et al. (2020) to consider dams of different dimensions, densities and locations. This is crucial as the numbers, densities and size of dams represented by Neumayer et al. (2020) are relatively small at the catchment scale in comparison to those observed or predicted elsewhere (Graham et al., 2020; Macfarlane et al., 2017; Zavyalov, 2014). Once greater confidence in estimating flow attenuation at extreme flows is gained, it will be important to consider how to extrapolate these findings to the catchment scale. By combining our understanding of where beavers build dams and in what densities (Dittbrenner et al., 2018; Graham et al., 2020; Hartman & Tornlov, 2006; Macfarlane et al., 2017; Swinnen et al., 2019), alongside an estimate of the inundated area that may occur (Karran et al., 2016), and an understanding of how flow attenuation manifests across varying event magnitudes (Neumayer et al., 2020; Nyssen et al., 2011; Puttock et al., 2021; Westbrook et al., 2020), flow states (Ronnquist & Westbrook, 2021; Woo & Waddington, 1990) and hydrometric conditions (Majerova et al., 2015; Westbrook et al., 2020), it will be possible to build a much stronger understanding of the catchment scale hydrological impacts of beaver.

Multiple studies now demonstrate the local scale impact of beaver dams on hydrology (Nyssen et al., 2011; Puttock et al., 2021, 2017), but there is a lack of empirical work that considers hydrological change at the (sub)catchment scale. Modelling that attempts landscape‐scale extrapolation of local impacts would greatly benefit from empirical work also conducted at this scale (Brazier, Elliott, et al., 2020; Brazier, Puttock, et al., 2020; Larsen et al., 2021). Dam sequences have already been shown to exert a larger effect on hydrology than single dams (Beedle, 1991), it is therefore reasonable to assume that this cumulative effect may also prevail when considering the impact of multiple dam complexes within a catchment, though it is not yet proven to what extent this may manifest. As shown for woody debris dams (Dixon et al., 2016; Lane, 2017), there is likely to be a cumulative effect, but this is unlikely to simply equate to the sum of the impact of individual dam complexes (Larsen et al., 2021). This understanding will prove key for informing future policy on beaver management but also effective approaches for human‐engineered NFM projects that seek to replicate beaver dam processes (Auster et al., 2022; Munir & Westbrook, 2021).

## 5 CONCLUSION

This study provides further evidence that beaver dam sequences attenuate peak flows during hydrological events. Peak flow attenuation increased with total event rainfall but there was considerable uncertainty for events where total rainfall was >97th percentile. The process of attenuation was demonstrated through the analysis of GAM hydrographs which showed clear changes in hydrograph geometry, following beaver dam complex construction, with increased lag time and reduced rising limb slope. Transient floodplain storage is likely to play a more significant role in contributing to the observed attenuation in addition to pond storage, groundwater losses and reduced velocity, resulting from an increase in roughness and decrease in channel slope and an increase in the area over which hydrological floodplain processes may occur. It is suggested that substantive transient floodplain storage may only occur in streams with low‐profile floodplain valleys and therefore these stream reaches are likely to yield the most substantive attenuation effect. The impacts on hydrological regime were most apparent during hydrological extremes—both high and low flows; changes to the frequency and magnitude of intermediate flows were negligible. Spatial, geographic, and meteorological variability will play a major role in determining the relative importance of attenuation mechanisms at play in beaver wetlands.

This research has important implications for beaver reintroduction and management. Beavers may contribute to flood resilience strategies such as natural flood management and catchment restoration, where dams occur in landscapes that support the transient flow attenuation mechanisms discussed herein. In these locations, beaver dam complexes may offer some low‐cost flood resilience to small, at‐risk communities, especially of value where conventional flood‐risk solutions may not be financially justified. The potential cumulative effect of many hundreds of dams could also have significant implications for catchment scale hydrological processes and thus flood‐risk reduction, but a stronger understanding of the spatio‐temporal variability in beaver dam‐hydrological interactions is needed to quantify such effects.

## Supporting information


**Appendix S1**. Supporting Information.

## Data Availability

All code and data to reproduce this analysis is available from the following repository https://doi.org/10.5281/zenodo.6034308 (Graham, 2022). The code used to download and extract The Met Office NIMROD rainfall radar time series (Met Office, 2003) can be found in this repository: https://github.com/exeter-creww/Rainfall_radar.
