# Supplementary material for: Exploring the dynamics of flow attenuation at a beaver dam sequence
Source: Hydrol Process. 2022 Nov 1;36(11):e14735. doi: 10.1002/hyp.14735 (PMC9828392; doi:10.1002/hyp.14735)
Supplement: Supplementary file 1 — Appendix S1. Supporting Information. [file HYP-36-0-s001.pdf]

Supplementary Information (SI):

SI 1: **A** - Hayes Lane gauging station and weir at East Budleigh. **B**- Location of flow gauge installation ~50 m upstream of the weir pictured in panel A. The gauge remained in situ for 2 months to measure flows, allowing for the creation of a rating curve to estimate flow from the depth gauge downstream.

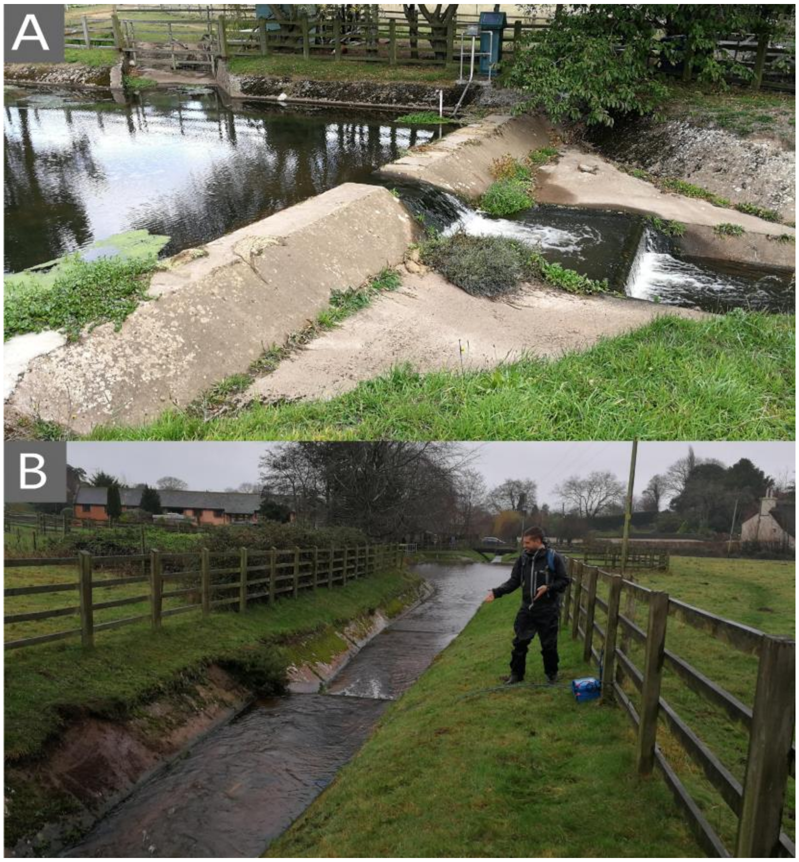

SI 2: stage-discharge rating using spline regression for Budleigh Brook EA gauging station. Upper plot shows the curve fit for the measured flow range. Lower plot shows extrapolation of curve to maximum and minimum flow limits from full time series. The equation denotes the piecewise polynomials that describe the curve.

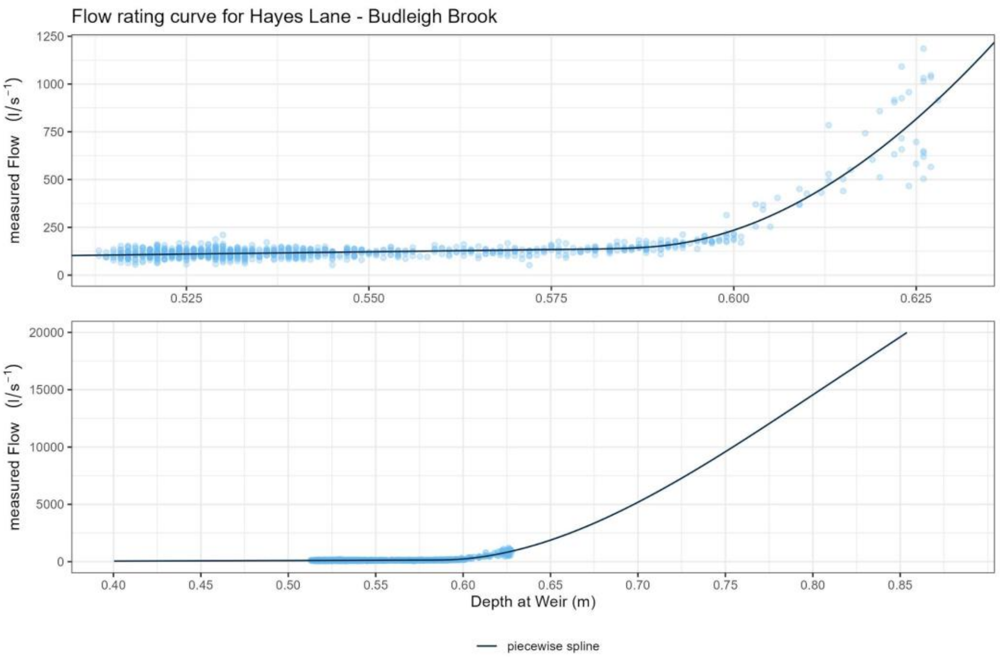

Piecewise polynomials of degree 3:
*y = 7.24e-14 + 474 * (x - 0.58) + 4.69e-08 * (x - 0.58) ^ 2 + 1.17e+07 * (x - 0.58) ^ 3*
*y = 31.9 + 6.42e+03 * (x - 0.593) + 4.57e+05 * (x - 0.593) ^ 2 - 7.36e+05 * (x - 0.593) ^ 3*

SI 3: Hydrological time series for Budleigh and Colaton Brook.

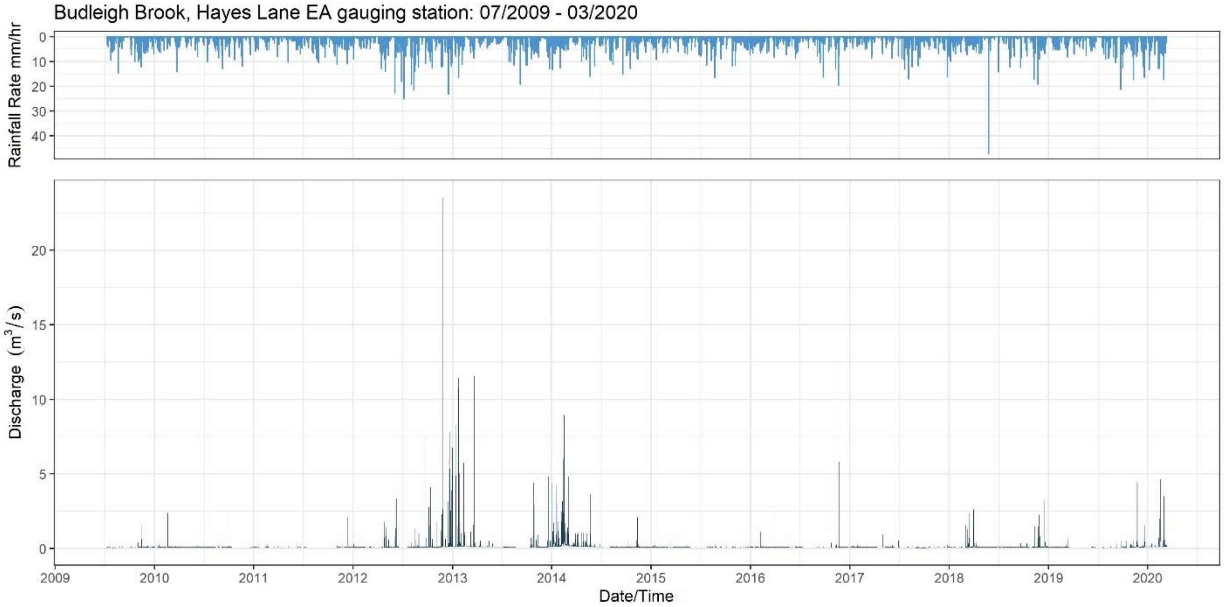

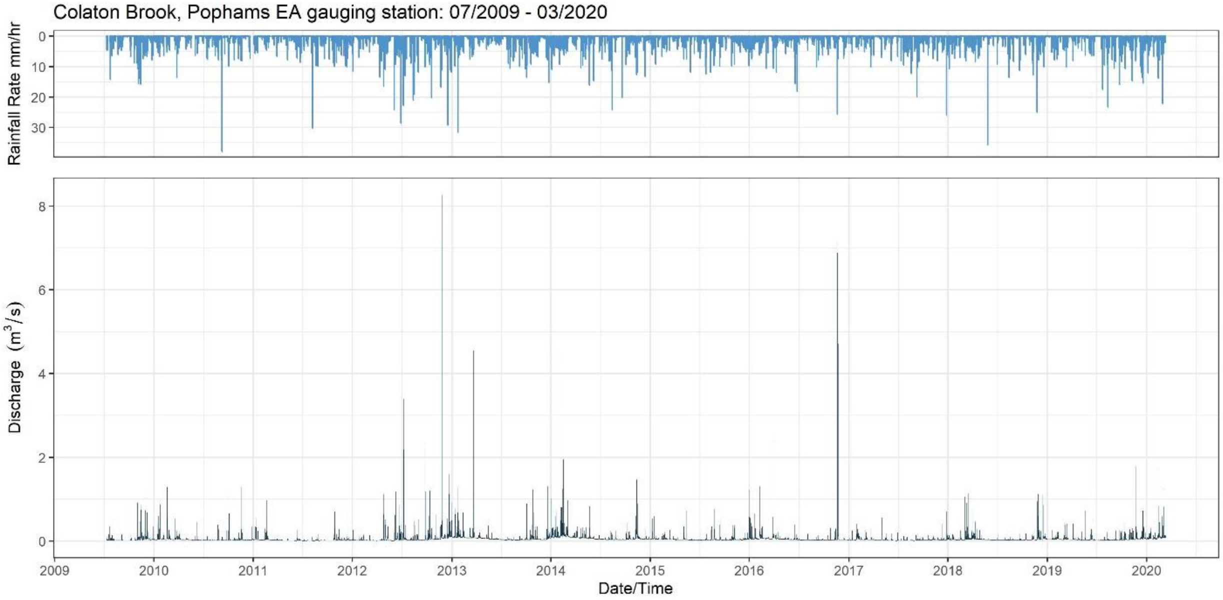

SI 4: Schematic describing the method used to automatically extract discrete hydrological events. From Puttock et al., (2021).

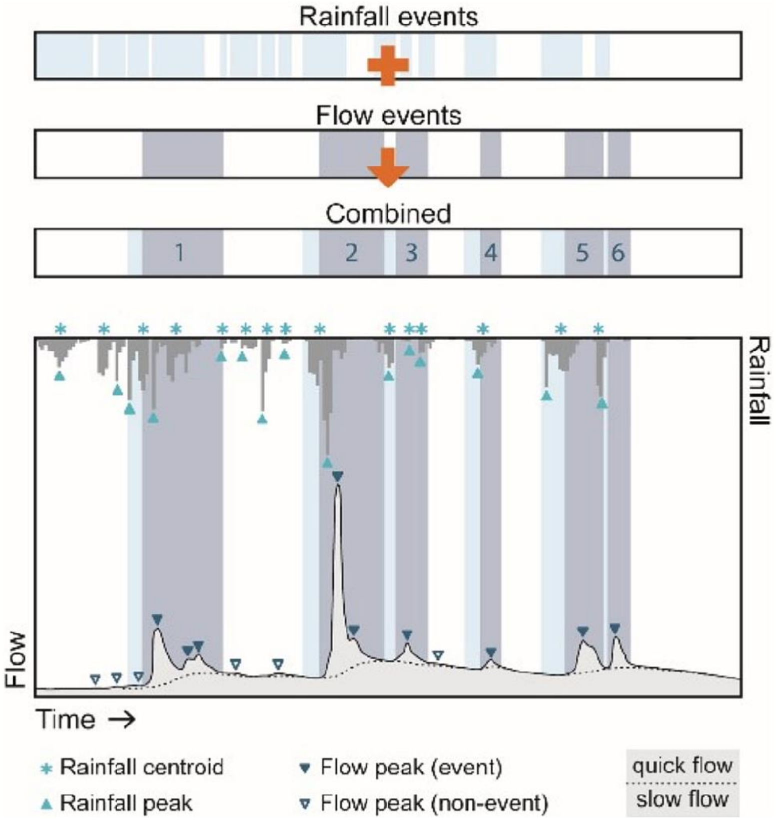

SI 5: Raincloud Plot showing the raw data, boxplot statistics and density distribution of peak flows for hydrological events in both Budleigh Brook and Colaton brook, before and after beaver complex was established in Budleigh Brook

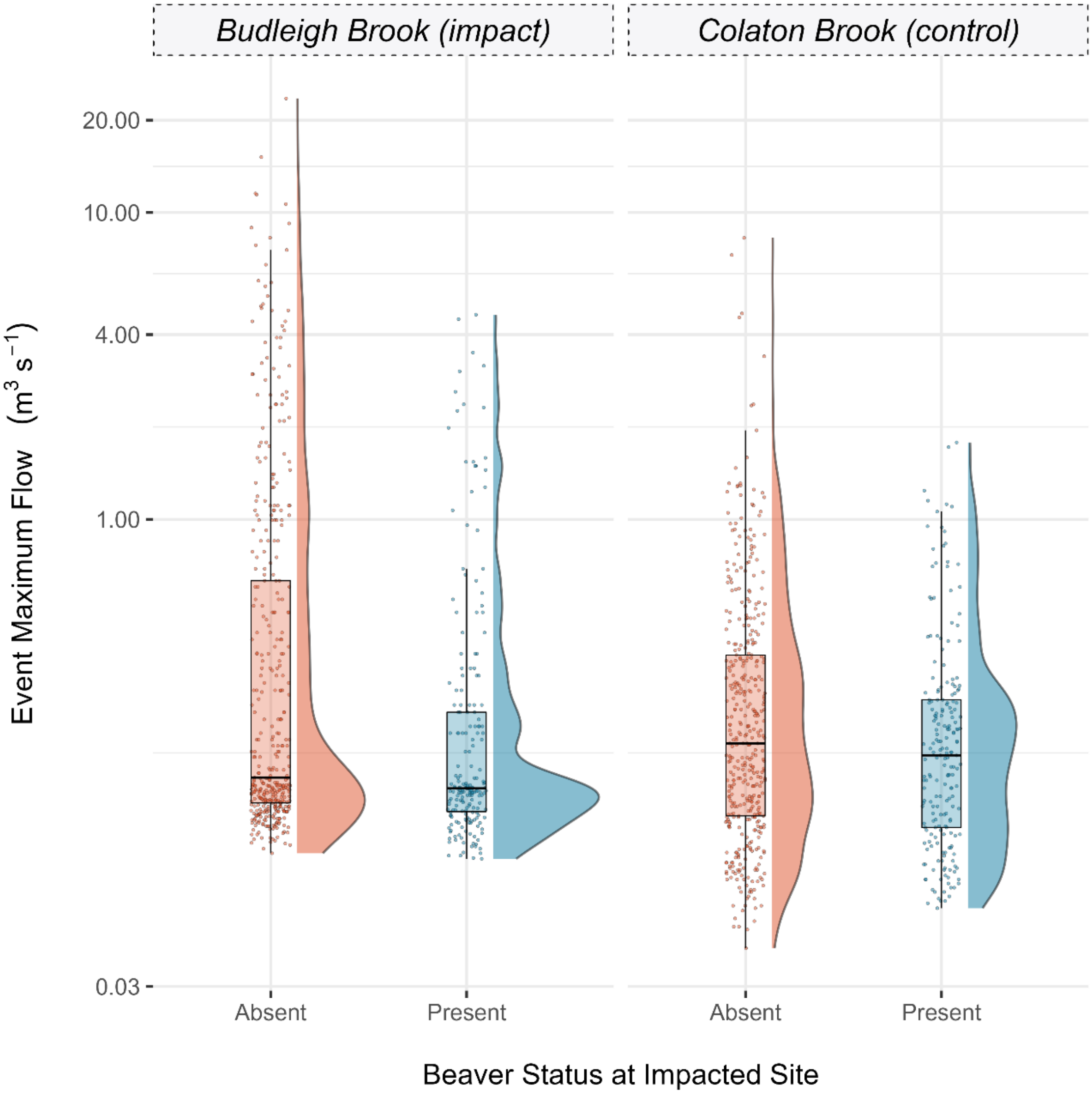

SI 6: General Linear Model validation plots produced using the {performance} R package (Lüdecke et al., 2021). The key diagnostic in these figures is the residual plots in the upper left; here, heteroscedasticity is not of such importance because we have adopted a Gamma error distribution. Critically, we are interested in the deviance in the fitted line through the residuals. It is clear that in M1 and M2, there is considerable skew in this line, indicating that the model is not appropriately describing the trend in the data, particularly for higher predicted flows. For M3, we some improvement but there is generally a poorer model fit. M4 and M5 provide better fit for larger predictions with less deviation in the trend line – of the two M5 provides the least deviation in the trend line, especially for larger values. Heavy tails in the bottom right (normality of residuals or QQ plot) indicate that for all models, there is likely to be some uncertainty in the predicted standard errors for extreme values.

Linear Additive (Identity-link)

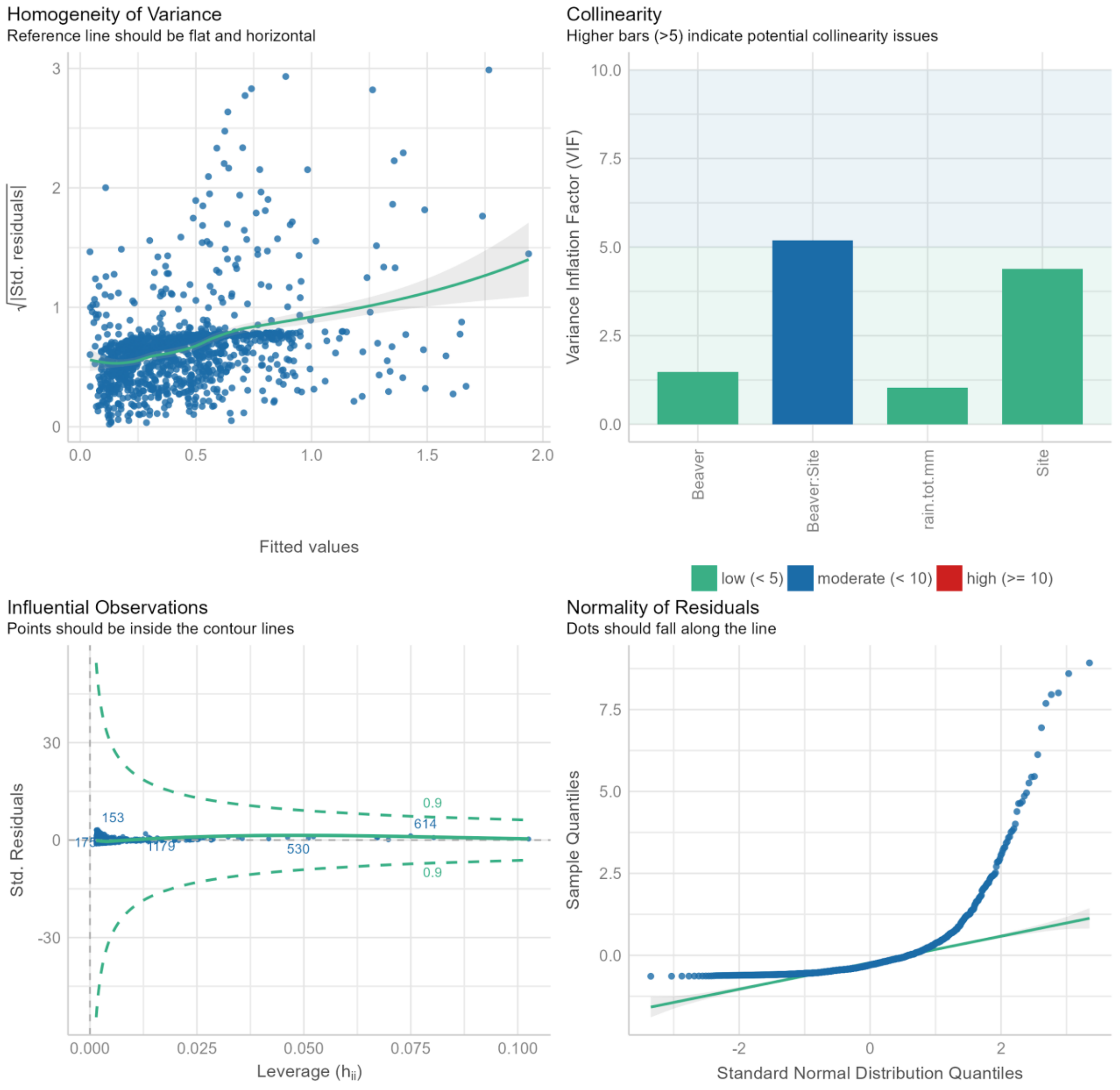

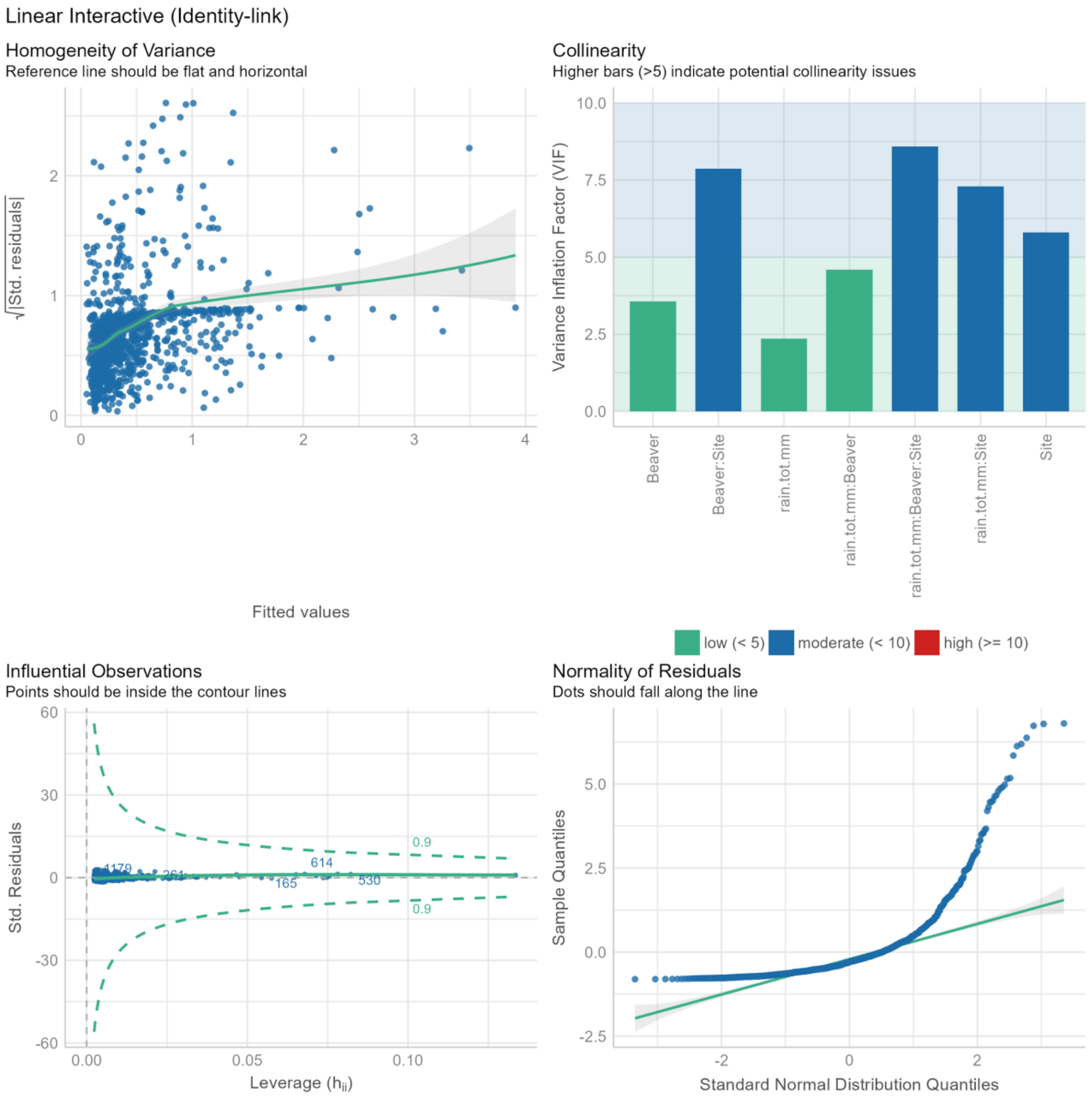
Linear Interactive (Identity-link)
Homogeneity of Variance
Reference line should be flat and horizontal
√|Std. residuals|
Fitted values
Collinearity
Higher bars (>5) indicate potential collinearity issues
Variance Inflation Factor (VIF)
Beaver
Beaver:Site
rain.tot.mm
rain.tot.mm:Beaver
rain.tot.mm:Beaver:Site
rain.tot.mm:Site
Site
low (< 5)
moderate (< 10)
high (>= 10)
Influential Observations
Points should be inside the contour lines
Std. Residuals
Leverage (h_{ii})
Normality of Residuals
Dots should fall along the line
Sample Quantiles
Standard Normal Distribution Quantiles

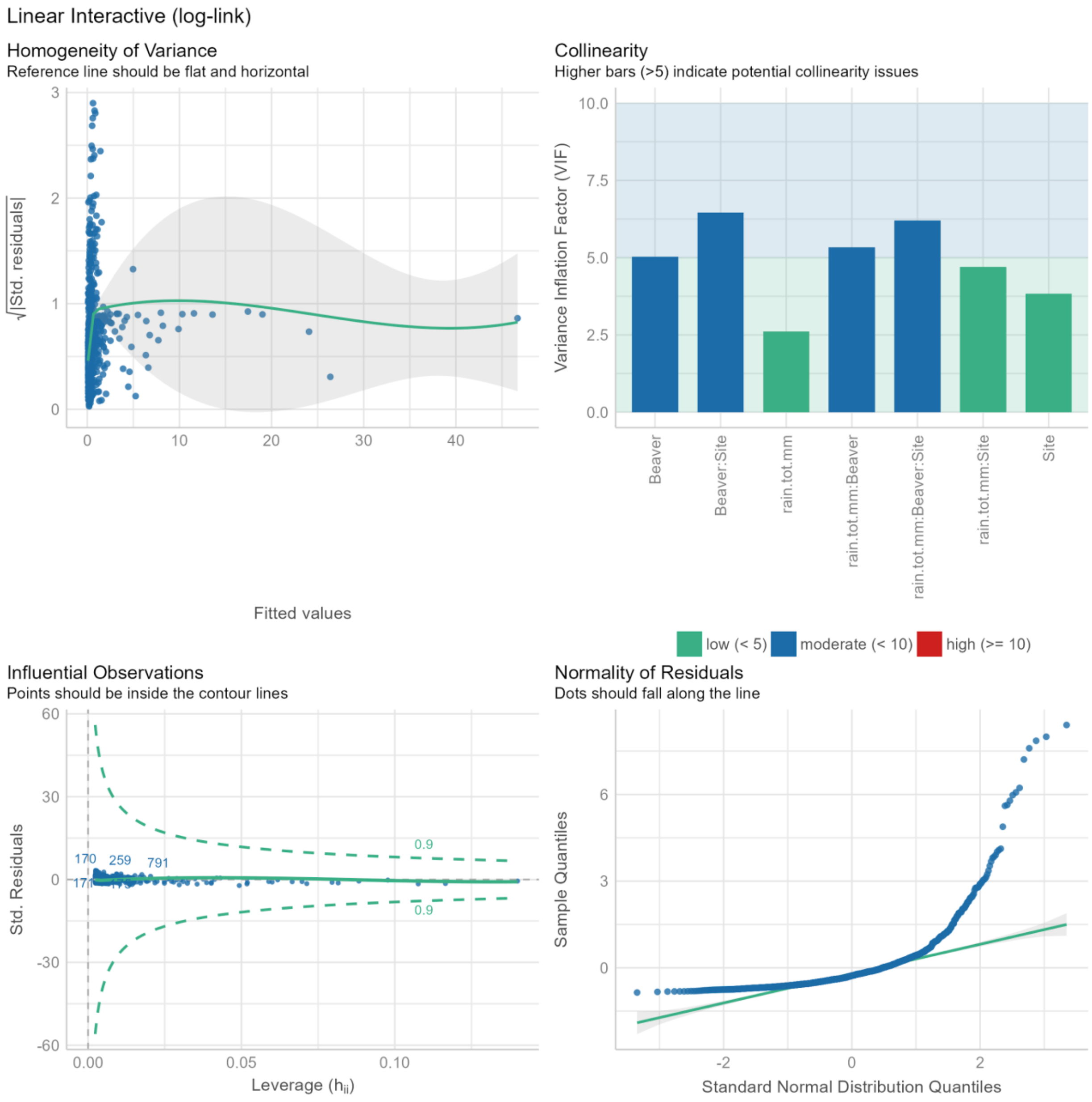
Linear Interactive (log-link)
Homogeneity of Variance
Reference line should be flat and horizontal
√|Std. residuals|
Fitted values
Collinearity
Higher bars (>5) indicate potential collinearity issues
Variance Inflation Factor (VIF)
Beaver
Beaver:Site
rain.tot.mm
rain.tot.mm:Beaver
rain.tot.mm:Beaver:Site
rain.tot.mm:Site
Site
low (< 5)
moderate (< 10)
high (>= 10)
Influential Observations
Points should be inside the contour lines
Std. Residuals
Leverage (h_{ii})
170
259
791
0.9
0.9
Normality of Residuals
Dots should fall along the line
Sample Quantiles
Standard Normal Distribution Quantiles

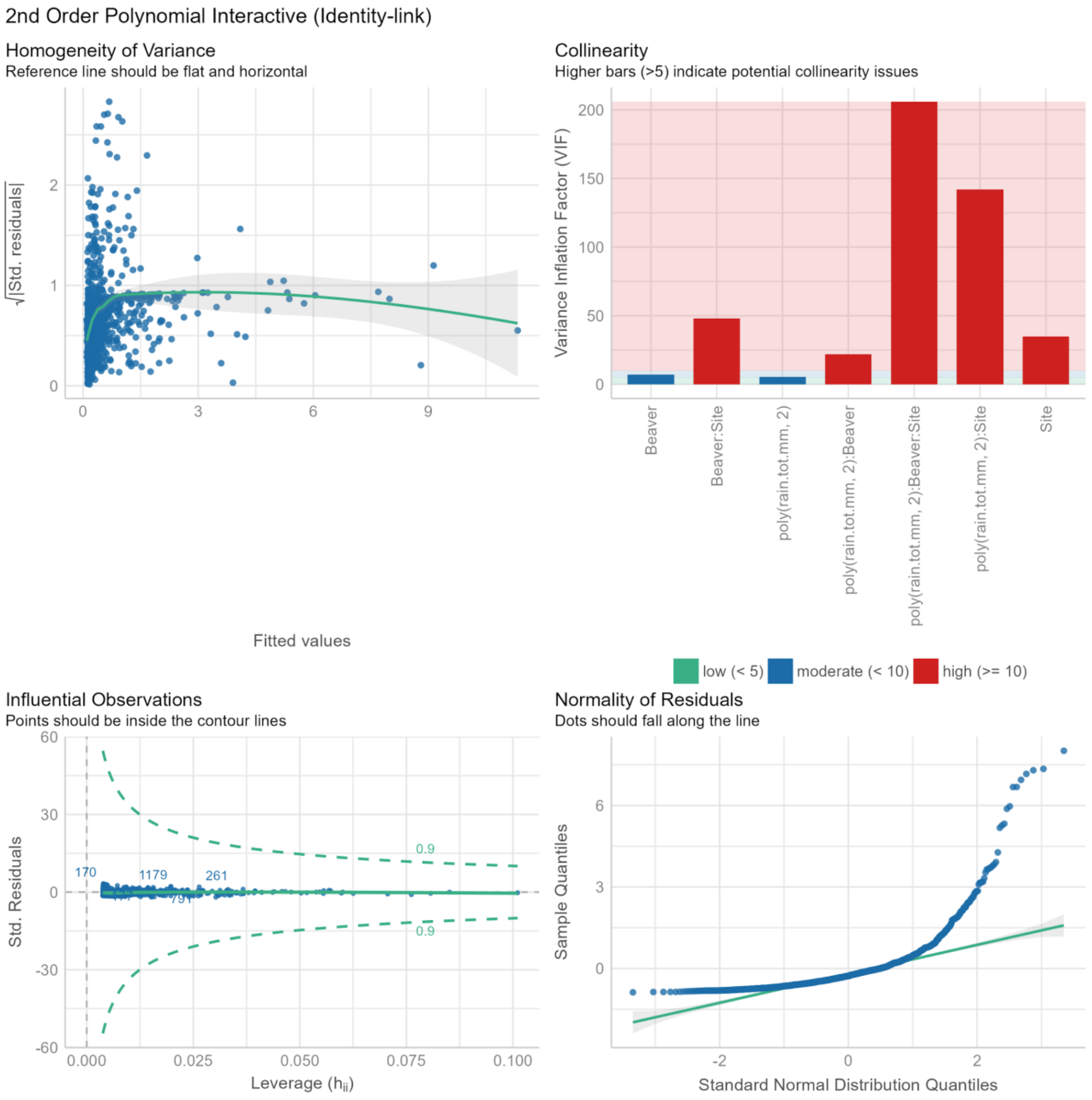
2nd Order Polynomial Interactive (Identity-link)
Homogeneity of Variance
Reference line should be flat and horizontal
√|Std. residuals|
Fitted values
Collinearity
Higher bars (>5) indicate potential collinearity issues
Variance Inflation Factor (VIF)
Beaver
Beaver:Site
poly(rain.tot.mm, 2)
poly(rain.tot.mm, 2):Beaver
poly(rain.tot.mm, 2):Beaver:Site
poly(rain.tot.mm, 2):Site
Site
low (< 5)
moderate (< 10)
high (>= 10)
Influential Observations
Points should be inside the contour lines
Std. Residuals
Leverage (h_{ii})
Normality of Residuals
Dots should fall along the line
Sample Quantiles
Standard Normal Distribution Quantiles

2nd Order Polynomial Interactive (log-link)

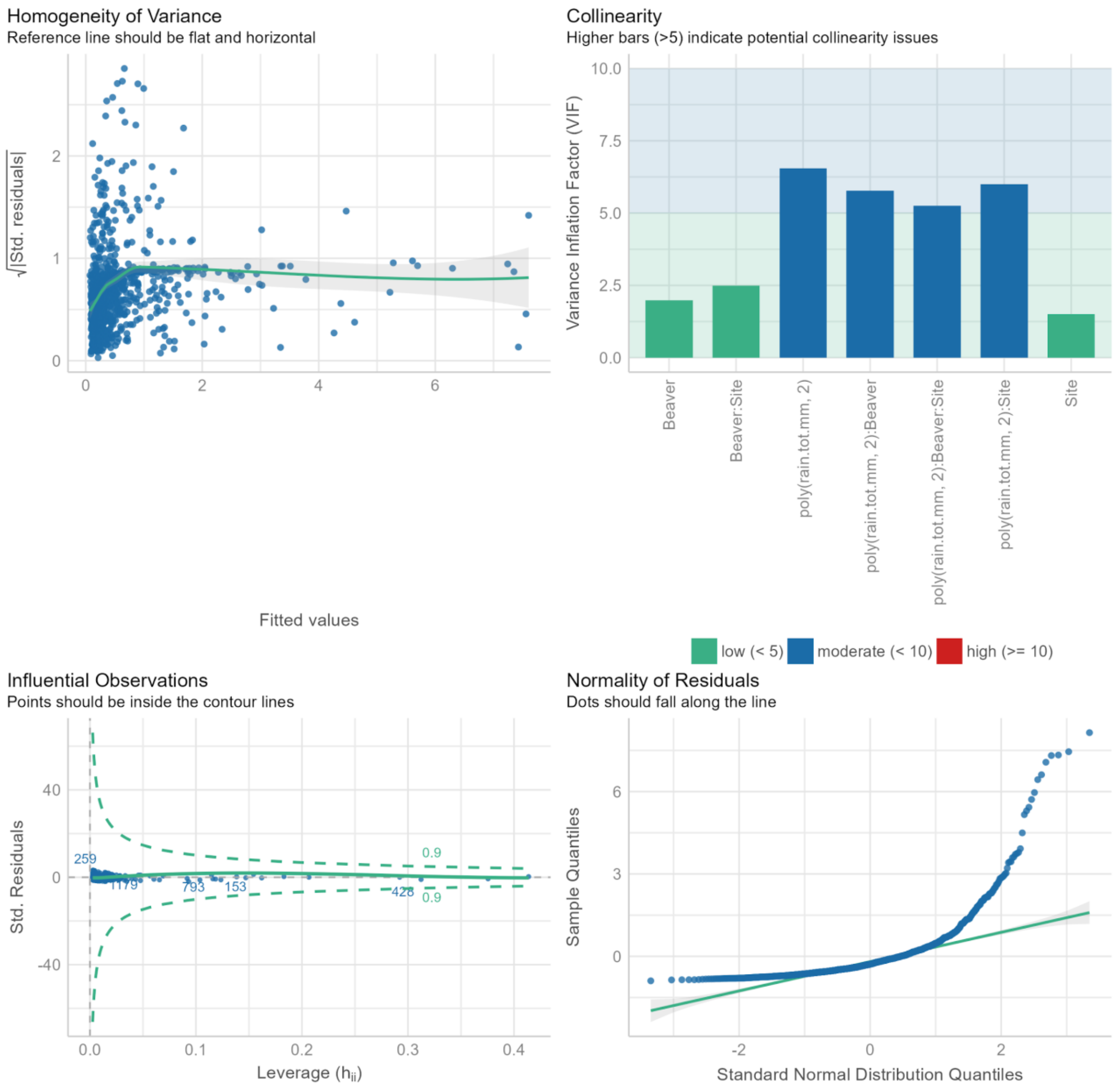

SI 7: General Linear Model Summary tables for the five general linear model presented in Figure 5. Corresponding Model evaluation plots are presented in SI6.

M1 Linear Add.

term	estimate	std.error	T.statistic	p.value
**(Intercept)**	0.042	0.016	2.641	0.008 *
**rain.tot.mm**	0.025	0.002	12.567	< 0.001 **
**BeaverPresent**	0.000	0.021	0.016	0.987
**SiteBudleigh Brook (impact)**	0.326	0.042	7.766	< 0.001 **
**BeaverPresent:SiteBudleigh Brook (impact)**	-0.315	0.049	-6.418	< 0.001 **

M2 Linear Int.

term	estimate	std.error	T.statistic	p.value
**(Intercept)**	0.051	0.016	3.090	0.002 *
**rain.tot.mm**	0.023	0.002	9.471	< 0.001 **
**BeaverPresent**	0.030	0.028	1.069	0.285
**SiteBudleigh Brook (impact)**	0.058	0.039	1.516	0.130
**rain.tot.mm:BeaverPresent**	-0.009	0.004	-2.454	0.014 *
**rain.tot.mm:SiteBudleigh Brook (impact)**	0.039	0.007	5.923	< 0.001 **
**BeaverPresent:SiteBudleigh Brook (impact)**	-0.084	0.050	-1.662	0.097 .
**rain.tot.mm:BeaverPresent:SiteBudleigh Brook (impact)**	-0.028	0.008	-3.436	< 0.001 **

M3 Linear Int. (log-link)

term	estimate	std.error	T.statistic	p.value

**(Intercept)**	-2.168	0.091	-23.743	< 0.001 **
**rain.tot.mm**	0.070	0.006	12.064	< 0.001 **
**BeaverPresent**	-0.022	0.159	-0.141	0.888
**SiteBudleigh Brook (impact)**	0.793	0.131	6.075	< 0.001 **
**rain.tot.mm:BeaverPresent**	-0.015	0.010	-1.418	0.156
**rain.tot.mm:SiteBudleigh Brook (impact)**	0.014	0.009	1.581	0.114
**BeaverPresent:SiteBudleigh Brook (impact)**	-1.044	0.230	-4.547	< 0.001 **
**rain.tot.mm:BeaverPresent:SiteBudleigh Brook (impact)**	0.031	0.016	1.936	0.053 .

**M4 Poly. Int.**				
**term**	estimate	std.error	T.statistic	p.value
**(Intercept)**	0.342	0.025	13.771	< 0.001 **
**poly(rain.tot.mm, 2)1**	12.997	2.355	5.519	< 0.001 **
**poly(rain.tot.mm, 2)2**	4.608	1.683	2.739	0.006 *
**BeaverPresent**	-0.084	0.036	-2.338	0.020 *
**SiteBudleigh Brook (impact)**	0.631	0.086	7.362	< 0.001 **
**poly(rain.tot.mm, 2)1:BeaverPresent**	-4.741	3.405	-1.392	0.164
**poly(rain.tot.mm, 2)2:BeaverPresent**	-1.368	2.488	-0.550	0.583
**poly(rain.tot.mm, 2)1:SiteBudleigh Brook (impact)**	29.677	8.050	3.687	< 0.001 **
**poly(rain.tot.mm, 2)2:SiteBudleigh Brook (impact)**	11.093	5.247	2.114	0.035 *
**BeaverPresent:SiteBudleigh Brook (impact)**	-0.398	0.110	-3.634	< 0.001 **

**poly(rain.tot.mm, 2)1:BeaverPresent:SiteBudleigh Brook (impact)**	-8.101	10.333	-0.784	0.433
**poly(rain.tot.mm, 2)2:BeaverPresent:SiteBudleigh Brook (impact)**	1.544	6.788	0.227	0.820

**M5 Poly. Int. (log-link)**				
**term**	estimate	std.error	T.statistic	p.value
**(Intercept)**	-1.362	0.055	-24.812	< 0.001 **
**poly(rain.tot.mm, 2)1**	23.149	1.846	12.540	< 0.001 **
**poly(rain.tot.mm, 2)2**	-4.280	1.791	-2.390	0.017 *
**BeaverPresent**	-0.189	0.096	-1.967	0.049 *
**SiteBudleigh Brook (impact)**	0.950	0.078	12.110	< 0.001 **
**poly(rain.tot.mm, 2)1:BeaverPresent**	-5.441	3.280	-1.659	0.097 .
**poly(rain.tot.mm, 2)2:BeaverPresent**	0.738	3.162	0.233	0.815
**poly(rain.tot.mm, 2)1:SiteBudleigh Brook (impact)**	3.137	2.758	1.138	0.256
**poly(rain.tot.mm, 2)2:SiteBudleigh Brook (impact)**	-2.579	2.736	-0.943	0.346
**BeaverPresent:SiteBudleigh Brook (impact)**	-0.703	0.137	-5.138	< 0.001 **
**poly(rain.tot.mm, 2)1:BeaverPresent:SiteBudleigh Brook (impact)**	6.317	5.138	1.229	0.219
**poly(rain.tot.mm, 2)2:BeaverPresent:SiteBudleigh Brook (impact)**	-1.701	5.390	-0.316	0.752
